# LATTICE: a governance-first architecture for authorized autonomous AI operations

**DOI:** 10.3389/frai.2026.1800407

**Published:** 2026-08-14

**Authors:** Elias Calboreanu

**Affiliations:** Capitol Technology University, Laurel, MD, United States

**Keywords:** AI governance, autonomous agents, cryptographic audit, human oversight, multi-agent systems, policy enforcement, safety assurance, separation of concerns

## Abstract

Deploying autonomous AI agents in high-consequence operational environments requires organizational authorization, yet few frameworks provide end-to-end, testable governance mechanisms suitable for such authorization decisions. This paper introduces LATTICE (Layered Agentic Triad Topology for Intelligent Coordinated Execution), a governance-first architecture that reframes the authorization question from “do we trust this AI?” to “do we trust this architecture?” The latter question is answerable through engineering validation rather than assumptions about model behavior. LATTICE enforces separation of concerns across planning, execution, and governance functions through a 1+3 Grid Cell pattern, so that no single component can both decide actions and judge compliance. The architecture implements policy-as-code enforcement with deterministic verdicts, gated execution paths that, under stated trusted-infrastructure assumptions (A1–A5), prevent unauthorized actions, confidence-based escalation to human operators, and cryptographic audit trails that preserve complete decision provenance. Empirical results characterize the AEGIS reference implementation; architecture-level properties are analytic, under stated assumptions. The governance engine is released as open source and reproduces its core results on commodity hardware: deterministic verdicts with zero deviations across 13 configurations repeated 10,000 times each, and no bypass in a 21-vector adversarial suite (0/21 observed; one-sided 95% upper bound 13.3%). In a pre-specified, planner-invariant *safety* evaluation (not an autonomy benchmark) across four frontier planner families (GPT-5, Claude Sonnet 4.6, Gemini, Grok-4; 4,000 trajectories), a confidence-threshold baseline's false-allow rate ranged from 0.03 to 0.998 across planners, whereas the AEGIS reference implementation admitted zero unsafe actions (false-allow 0.0, recall 1.0) invariant to the planner, at a conservative operating point that auto-allowed no action; a separate live run additionally governed real operating-system actions with zero unsafe executions. Governance latency is low and host-specific (on an Apple M4 Pro: policy evaluation p50 ≈ 6.2 μs; full gated enforcement p50 ≈ 0.7 ms including audit I/O). LATTICE provides a pathway for responsible deployment of autonomous AI in defense, critical infrastructure, and regulated industries where authorization requires verifiable governance rather than trust in AI behavior.

## 1 Introduction

The emergence of large language model (LLM) based autonomous agents has created a fundamental tension: these systems demonstrate remarkable capability for complex task execution, yet organizations cannot authorize their deployment in high-consequence environments because no widely accepted principled basis exists for organizational authorization of autonomous LLM agents in such contexts. Defense organizations, critical infrastructure operators, and regulated industries face mounting pressure to adopt AI capabilities, but authorization requires confidence that autonomous systems will comply with legal constraints, organizational policies, and operational rules of engagement.

The urgency of this challenge is underscored by a rapidly growing body of real-world incidents. The AI Incident Database's ongoing incident roundups document a sustained rise in reported AI-related incidents through 2025 and into 2026, with autonomous agent failures representing an increasing proportion of serious cases (AI Incident Database, 2026). In July 2025, an AI coding assistant deleted 1,206 production database records in seconds, ignoring an active code freeze and refusing post-incident commands from the developer to cease further changes (AI Incident Database, 2025). In a separate incident, an expense-processing agent, unable to interpret receipts, fabricated plausible entries including fictitious restaurant names to meet its completion objective (Boston Consulting Group, 2025). A McKinsey survey found that 80% of organizations deploying AI agents have encountered risky or unexpected behavior (McKinsey & Company, 2025), while Boston Consulting Group warned that autonomous AI agents represent “the next era of risk” for enterprises (Boston Consulting Group, 2025). The December 2025 release of the OWASP Top 10 for Agentic Applications, the first formal taxonomy of risks specific to autonomous AI agents, developed by over 100 security researchers, codified threats including agent goal hijacking, tool misuse, identity abuse, memory poisoning, and rogue agent behavior (OWASP Foundation, 2025a). A systematic survey of 30 deployed agentic AI systems confirms that this governance deficit is structural: 25 of 30 agents disclose no internal safety evaluation results, and 23 of 30 have undergone no third-party testing (Staufer et al., 2025). These incidents and frameworks demonstrate that the governance gap LATTICE addresses is not theoretical but operationally urgent.

Current approaches to this challenge are inadequate. Systems that prioritize capability allow agents to operate without sufficient governance, creating unacceptable risks when unauthorized actions have operational, legal, or safety consequences. Systems that prioritize control impose human-approval requirements that undermine the efficiency benefits of automation. Both approaches treat autonomy and governance as opposing forces in a zero-sum tradeoff.

This paper introduces LATTICE (Layered Agentic Triad Topology for Intelligent Coordinated Execution), a governance-first architecture that resolves this tension by treating autonomy and governance as independently tunable properties. The core insight is architectural: rather than attempting to ensure AI systems behave correctly (a goal that cannot be reliably certified for probabilistic generative systems), LATTICE ensures the architecture constrains behavior to acceptable bounds through deterministic enforcement mechanisms.

This reframing transforms the authorization question. Instead of asking ‘do we trust this AI?' (a question that cannot be definitively answered given the opacity of LLM reasoning), organizations can ask ‘do we trust this architecture?' The latter question can be answered through engineering validation: scope enforcement can be verified, policy compliance can be audited, escalation thresholds can be tested, and halt mechanisms can be demonstrated. Authorization becomes an engineering problem rather than an act of faith. This is an architecture-and-systems contribution: LATTICE is evaluated by engineering-validation criteria (determinism, complete mediation, auditability, and reproducibility) rather than model-performance benchmarks, and its central guarantees are analytic properties of the architecture under stated assumptions rather than learned model behaviors.

This distinction requires precise terminology. Before proceeding, we clarify our use of ‘authorization' throughout this paper. We distinguish three forms: (1) legal authorization, meaning permission under applicable law, regulation, and contractual agreements; (2) organizational authorization, meaning explicit approval from institutional decision-makers with authority to accept operational risk; and (3) technical authorization, meaning access control mechanisms that permit or deny specific actions. LATTICE primarily addresses organizational authorization by providing the architectural basis for institutional decision-makers to approve autonomous operations with confidence in governance guarantees that are conditional on explicit, stated infrastructure assumptions (A1–A5, Section 6).

This paper addresses four research questions that guide the investigation:

- **RQ1:** How can autonomous agents operate continuously while remaining under enforceable human governance?
- **RQ2:** What architectural controls enable deterministic authorization decisions for probabilistic agents?
- **RQ3:** How can a system maintain safety and effectiveness simultaneously in adversarial environments?
- **RQ4:** What evidence artifacts are sufficient to demonstrate governance assurance to reviewers and auditors?

The contributions of this paper are:

1. A theoretical framework synthesizing separation of concerns, functional safety, and regulatory requirements to justify governance-first architecture design
2. The 1+3 Grid Cell pattern that enforces separation between planning, execution, and governance functions
3. Governance mechanisms including policy-as-code enforcement, deterministic enforcement boundaries, and cryptographic audit trails
4. Safety property statements with explicit assumptions, enforcement mechanisms, and empirically validated verification conditions
5. A confidence-based decision model that routes human oversight according to a policy-derived risk signal
6. A worked example demonstrating the complete authorization chain
7. A formal threat model with concrete attack scenarios and architectural defenses
8. Open, reproducible empirical validation of the AEGIS (Autonomous Engineering Governance and Intelligence System) reference implementation, spanning determinism, a 21-vector adversarial suite, threshold-sensitivity analysis, and host-disclosed latency, together with a pre-specified, planner-invariant *safety* evaluation across four frontier planner families (4,000 trajectories): a confidence-threshold authorization baseline's false-allow rate ranges from 0.03 to 0.998 across planners, whereas the reference implementation admits zero unsafe actions (false-allow 0.0, recall 1.0) invariant to the planner model, at a conservative operating point that auto-allowed no action (planner-invariant safety, not autonomy; the live closed-loop run of Section 9.12 partially exercises the autonomous path; full results in Section 9)
9. A structured comparative taxonomy positioning LATTICE against existing frameworks across architectural governance, model-level alignment, and framework-level orchestration categories

**Scope of claims**. We state the boundaries of what follows at the outset. The empirical results characterize a single reference implementation (AEGIS); the architecture-level properties (separation of concerns and non-bypassability) are analytic and hold conditionally on the trusted-infrastructure assumptions A1–A5 (Section 6), non-bypassability in particular on sandbox isolation (A5). The real-LLM evaluation establishes planner-invariant *safety* at a conservative operating point that auto-allowed no action; it is not an autonomy benchmark, and its ground truth is a specification-derived rule oracle that shares the engine's structured-attribute ontology. Latency figures are host-specific. The confidence score is a governance triage heuristic, not a calibrated estimator of real-world risk. Each of these bounds is developed where the corresponding result is presented.

## 2 Related work

LATTICE synthesizes insights from cognitive architectures, LLM agent patterns, functional safety engineering, human-autonomy teaming research, and emerging AI governance frameworks.

### 2.1 Cognitive architectures and agent theory

The Belief-Desire-Intention (BDI) architecture provides a foundational framework for separating mental states from action execution (Bratman, 1987; Rao and Georgeff, 1995; de Silva et al., 2020). BDI's distinction between what an agent believes, what it wants to achieve, and what it has committed to doing informs LATTICE's separation of planning intent from execution commitment. SOAR and ACT-R cognitive architectures demonstrate the value of layered control models with explicit memory and decision separation (Anderson et al., 2004; Laird, 2012), motivating the architectural isolation between LATTICE components.

Wooldridge (Wooldridge, 2009) established that multi-agent systems enable complex task completion beyond single-agent capabilities through coordination, but also identified failure modes including miscoordination, conflict, and collusion.

### 2.2 LLM agent patterns

Contemporary research on LLM-based agents establishes both capabilities and limitations. Wang et al. (2024) present a unified framework identifying essential agent modules for planning, memory, and action. The ReAct pattern validates interleaved reasoning and action patterns Yao et al., (2023). Xi et al. (2023) demonstrate that multi-agent cooperation patterns enable complex task completion through hierarchical and blackboard-based coordination.

Research on autonomous security testing illustrates the capability-governance gap. Fang et al. (2024) achieved 87% success exploiting one-day vulnerabilities with hierarchical multi-agent systems. Zhu et al. (2024) demonstrated that teams of LLM agents can exploit zero-day vulnerabilities, achieving up to 4.3x improvement over prior single-agent approaches in web application contexts. However, neither system implements governance mechanisms suitable for authorized operations. Hammond et al. (2025) provide a detailed taxonomy of multi-agent risks from advanced AI, identifying three failure modes, miscoordination, conflict, and collusion, and seven underpinning risk factors including emergent agency and multi-agent security, underscoring the need for architectural governance mechanisms that operate independently of agent cooperation.

### 2.3 Functional safety and standards

IEC 61508 establishes that safety functions must be independent from control functions in safety-related systems (IEC, 2010). This principle directly informs LATTICE's separation of governance from execution. DO-178C (RTCA, 2011) codifies analogous separation requirements for airborne software, mandating that safety-critical functions undergo independent verification proportional to their Design Assurance Level, a graduated assurance model that parallels LATTICE's tiered confidence thresholds across deployment contexts. ISO 13850 provides design principles for emergency stop mechanisms (ISO, 2015), informing LATTICE's multi-modal kill-switch implementation.

### 2.4 Regulatory frameworks

The European Union AI Act (Regulation 2024/1689) mandates that high-risk AI systems allow effective human oversight, including the ability to understand outputs, monitor operations, and intervene when necessary (European Parliament and Council of the European Union, 2024). The NIST AI Risk Management Framework establishes that oversight measures should be commensurate with risk (Tabassi, 2023). The NIST Zero Trust Architecture (Rose et al., 2020) establishes that no implicit trust should be granted to assets or accounts based on network location alone, requiring continuous authentication and authorization as discrete functions before resource access, a principle that LATTICE enforces at the agent-action level through per-action policy evaluation and cryptographic verdict binding. The OWASP Top 10 for Agentic Applications (OWASP Foundation, 2025a) provides the first formal taxonomy of risks specific to autonomous AI agents, identifying ten categories including agent goal hijacking, tool misuse, identity abuse, and rogue agent behavior; Section 8.1 maps each risk to the LATTICE mechanism that addresses it. The companion OWASP Top 10 for LLM Applications (OWASP Foundation, 2025b) identifies Excessive Agency as a top risk, decomposing it into excessive functionality, permissions, and autonomy, dimensions that LATTICE constrains through policy-as-code, per-action authorization, and confidence-based escalation respectively. LATTICE operationalizes these requirements as architectural constraints.

### 2.5 Human-autonomy teaming

Research on human-autonomy teaming demonstrates that high automation and high human control can be achieved simultaneously through appropriate design (Shneiderman, 2022). Parasuraman et al. (2000) established levels of automation that inform confidence-based mode switching. Chen and Barnes (2014) demonstrate effective patterns for human-agent teaming in multi-agent control contexts.

### 2.6 AI governance approaches

LATTICE's policy-as-code approach builds on decades of access control research. Role-Based Access Control (RBAC) established that authorization decisions should be mediated by policy abstractions rather than hard-coded into application logic (Sandhu et al., 1996). Attribute-Based Access Control (ABAC) generalized this to dynamic, context-sensitive evaluation of subject, object, and environmental attributes against declarative rules (Hu et al., 2014). The eXtensible Access Control Markup Language (XACML) formalized declarative policy specification with architecturally separated Policy Decision Points (PDPs) and Policy Enforcement Points (PEPs) (OASIS, 2013), a pattern that LATTICE inherits through its separation of the Governance Agent (policy evaluation) from the Execution Gate (enforcement). These foundations establish that externalized, declarative policy evaluation is both more auditable and more composable than embedded authorization logic.

Existing AI governance approaches operate at three distinct levels, each addressing a different aspect of the safety challenge. LATTICE occupies the architectural level, which is complementary to (not competitive with) the other two.

**Model-level approaches** attempt to shape what the AI will do. Constitutional AI (CAI) (Bai et al., 2022) trains models to follow behavioral principles through reinforcement learning from AI feedback (RLAIF). The model internalizes constraints during training, producing outputs that are statistically more aligned with specified principles. However, CAI provides no deterministic guarantee that a specific harmful action will be blocked at inference time. The model may comply 99.9% of the time yet fail unpredictably on novel inputs, because compliance is an emergent property of training rather than an enforced invariant. Reinforcement Learning from Human Feedback (RLHF) (Ouyang et al., 2022) shares this limitation: alignment is probabilistic and can degrade under distribution shift. (Liu et al. 2026) introduce AgentDoG, a diagnostic guardrail that uses LLM-based models (4B–8B parameters) to monitor full execution trajectories and diagnose root causes of unsafe actions using a three-dimensional risk taxonomy; however, because the guardrail itself is probabilistic, it cannot provide the deterministic enforcement guarantees that architectural approaches offer. More broadly, techniques such as retrieval-augmented generation, self-reflection, and chain-of-thought prompting address the *accuracy problem*, reducing the probability of incorrect or hallucinated outputs, whereas LATTICE addresses the orthogonal *authorization problem*: admitting only policy-compliant actions, regardless of output quality. Risks such as unauthorized data exfiltration or out-of-scope tool invocation persist even when model outputs are factually correct, because they stem from insufficient action-level governance rather than insufficient reasoning capability.

**Framework-level approaches** provide tooling for building agents but delegate governance to the developer. AutoGPT (Significant Gravitas, 2023) implements a recursive self-prompting architecture where the agent autonomously generates and executes task chains. It includes no governance layer; the agent has unrestricted tool access and no mechanism for independent policy evaluation. LangChain (LangChain, Inc., 2023) provides agent abstractions with optional callback-based guardrails, but the guardrails are advisory middleware that the agent framework can bypass through direct tool invocation. CrewAI (Moura, 2024) introduces role-based multi-agent coordination with hierarchical processes, but governance remains embedded within agent prompts rather than enforced through architectural separation. Microsoft AutoGen (Wu et al., 2023) enables multi-agent conversations with configurable human-in-the-loop patterns, but does not implement deterministic policy evaluation or non-bypassable execution gates.

**Architectural-level approaches** constrain what the AI can do regardless of its internal reasoning. Wang et al. (2026) introduce AgentSpec, a domain-specific language for specifying and enforcing runtime constraints on LLM agents, achieving over 90% unsafe execution prevention in code agents and 100% compliance in the autonomous vehicle domain. AgentSpec demonstrates the viability of external runtime enforcement; however, it relies on rule-matching rather than deterministic policy evaluation, and does not provide cryptographic audit trails or formal non-bypassability guarantees. Time-of-check to time-of-use (TOCTOU) vulnerabilities, where validated state changes between verification and use, represent a foundational class of race conditions in security-critical systems (Bishop and Dilger, 1996). Lilienthal and Hong (2025) recently demonstrated that TOCTOU vulnerabilities affect LLM-enabled agents through malicious configuration swaps and payload injection between planning and execution phases, underscoring the need for deep-copy isolation at the governance boundary. Syros et al. (2025) propose SAGA, a security architecture for governing agentic systems through provider-based agent registration and cryptographically derived access control tokens with formal security guarantees; however, SAGA's centralized Provider model does not enforce TOCTOU-safe deep-copy isolation or deterministic policy evaluation at the individual action level. Gaurav et al. (2025) propose Governance-as-a-Service (GaaS), a modular policy-driven enforcement layer that regulates agent outputs at runtime using declarative rules and a Trust Factor mechanism for graduated enforcement; however, GaaS operates at the output-filtering level rather than the action-authorization level and lacks cryptographic audit chains for non-repudiation. Madan (2025) introduces ArGen, which combines Group Relative Policy Optimization (GRPO) with an OPA-inspired policy-as-code engine to align LLM behavior with configurable rule sets; unlike LATTICE, ArGen modifies model weights during training rather than enforcing policy externally at runtime, making it a hybrid model-level/architectural approach. Xu et al. (2025) present ArbiterOS, a governance-first approach that reframes the LLM as a “Probabilistic CPU” managed by a deterministic Governor through a formal Agent Constitution Framework; while ArbiterOS provides a compelling theoretical foundation with its neuro-symbolic operating system metaphor, it does not report empirical benchmarks or a reference implementation. Ge (2026) proposes a four-layer Layered Governance Architecture (LGA) comprising execution sandboxing, intent verification, zero-trust inter-agent authorization, and immutable audit logging, evaluated on a 1,081-sample bilingual benchmark achieving 93.0–98.5% interception rates; however, LGA relies on LLM-based intent judges at its verification layer, incurring p50 latency of approximately 980ms compared to LATTICE's deterministic policy evaluation at 6.2 μs (Section 9.8). Wang et al. (2025) present MI9, an integrated runtime governance framework that introduces six components, an agency-risk index, agent-semantic telemetry, continuous authorization monitoring, finite-state-machine (FSM)-based conformance engines, goal-conditioned drift detection, and graduated containment, for real-time oversight of agentic systems; MI9 addresses runtime behavioral drift that pre-deployment governance cannot anticipate, but its FSM-based conformance approach does not provide the deterministic, content-addressed policy evaluation or cryptographic audit chain that LATTICE guarantees. Datta et al. (2025) survey the emerging field of agentic AI security, identifying governance-centric and signal-centric enforcement approaches and cataloging threats spanning prompt injection, tool misuse, and privilege escalation; LATTICE aligns with their governance-centric approach while providing concrete architectural mechanisms absent from the survey's threat taxonomy. Bhardwaj (2026) introduces Agent Behavioral Contracts (ABC), bringing Design-by-Contract principles to autonomous agents through a four-tuple specification of preconditions, invariants, governance policies, and recovery mechanisms enforced at runtime; while ABC provides a principled contract-based formalism evaluated across 1,980 sessions on seven models, it operates at the behavioral specification level rather than enforcing non-bypassable architectural isolation between governance and execution. Kaptein et al. (2026) formalize runtime governance as deterministic compliance functions over agent execution paths, arguing that the path, not the individual action, is the central object for governance; their framework complements LATTICE's per-action authorization by providing a formal basis for reasoning about sequential policy compliance, though it does not address cryptographic auditability or TOCTOU isolation. Mavračić (2025) proposes Policy Cards, a machine-readable standard for encoding operational, regulatory, and ethical constraints as deployment-layer artifacts with crosswalk mappings to the NIST AI RMF, ISO/IEC 42001, and the EU AI Act; Policy Cards address the policy *specification* layer that LATTICE's policy-as-code bundles operationalize at the *enforcement* layer. Wu et al. (2025) present IsolateGPT, an execution isolation architecture published at NDSS that applies process-level sandboxing to LLM agent apps, defending against app compromise and data exfiltration through well-defined inter-app interfaces; IsolateGPT addresses the *execution isolation* dimension that complements LATTICE's *policy enforcement* dimension, but does not implement deterministic policy-as-code evaluation or cryptographic audit trails. Fatmi (2026) introduces Faramesh, a protocol-agnostic execution control plane whose Action Authorization Boundary (AAB) enforces deterministic authorization over canonical action representations with cryptographic hashing, supporting MCP, A2A, and ANP protocols; Faramesh shares LATTICE's core design principles of non-bypassability, determinism, and replayability, but does not provide tiered confidence-based human escalation, formal threat modeling, or the multi-agent supervisory architecture (Grid Cell, Oversight Matrix) that LATTICE uses to manage agent-to-human handoff. Abaev et al. (2026) propose AgentGuardian, which learns context-aware access-control policies from execution traces during a staging phase and enforces them at runtime based on multi-step control flow dependencies; while AgentGuardian adapts to application-specific behaviors, its learned policies are inherently probabilistic and cannot provide the deterministic, replay-verifiable verdicts that LATTICE's policy-as-code evaluation guarantees. LATTICE operates at this level, enforcing governance through structural separation rather than behavioral training or advisory middleware. The Execution Agent cannot access tools except through the Governance-controlled gate, and the gate requires an auditable ALLOW verdict bound to an immutable bundle hash before permitting any action. This transforms authorization from a probabilistic expectation to a verifiable engineering property.

The distinction is fundamental: model-level and framework-level approaches reduce the probability of harmful actions, while architectural enforcement constrains, by construction and under the stated trusted-infrastructure assumptions, entire categories of unauthorized action. The approaches are orthogonal and can be combined. An agent using a CAI-trained model within a LATTICE-governed architecture benefits from both probabilistic alignment and deterministic enforcement. A complementary line of work translates high-level, LLM-generated intent into explicit, tunable coordination policies for multi-agent systems (de Curtó et al., 2026). Where such parameterized-cooperation approaches shape *how* agents cooperate at the coordination layer, LATTICE governs *whether* each individual action is authorized, adding deterministic per-action authorization with fail-closed verdicts and cryptographic per-action auditability; the two are composable, since a parameterized cooperation policy can set the coordination intent that LATTICE's Grid Cells then enforce and audit action-by-action.

### 2.7 Comparison with existing frameworks

Table 1 presents a qualitative feature comparison across enforcement dimensions, and Table 2 provides a quantitative comparison using metrics as reported by each system's authors. We note that these systems target different domains, use different evaluation methodologies, and were evaluated under different conditions; the comparison illustrates architectural coverage differences rather than establishing strict performance rankings. Because this set draws heavily on 2025–2026 preprints and technical reports alongside peer-reviewed venues, each comparison source is classified by publication status in the Supplementary material. We define the comparison criteria:

- **Separation of duties**: Whether governance evaluation and action execution are performed by architecturally isolated components (not just logically distinct code paths).
- **Policy-as-code**: Whether governance rules are expressed as deterministic, machine-evaluable specifications rather than natural language guidelines or model prompts.
- **Non-bypassable gate**: Whether tool execution requires passage through a gate that verifies an auditable ALLOW verdict bound to an immutable content hash. “Non-bypassable” means the agent has no code path to tools that does not traverse the gate.
- **Deterministic verdicts**: Whether identical inputs always produce identical governance decisions, enabling replay verification.
- **Cryptographic audit**: Whether every governance decision produces a tamper-evident record linked by cryptographic hashes and digital signatures.
- **Kill switch**: Whether the architecture provides an independent halt mechanism that operates regardless of agent state or cooperation.
- **Formal threat model**: Whether the system provides a structured analysis of adversarial scenarios with identified threats, attack vectors, and corresponding architectural defenses.
- **Human escalation**: Whether the system includes a structured mechanism for routing low-confidence or policy-ambiguous decisions to human operators, as opposed to manual intervention.

**Table 1:** Feature comparison: LATTICE vs. established behavioral and advisory agent frameworks, based on each framework's published description as of the cited works.

Feature	LATTICE	Const. AI	AutoGPT	LangChain	CrewAI	AutoGen
Separation of duties	Enforced	No	No	No	Partial	Partial
Policy-as-code	Deterministic	No	No	Partial	No	No
Non-bypassable gate	Yes	No	No	No	No	No
Deterministic verdicts	Yes	No	No	No	No	No
Cryptographic audit	Hash chain + ECDSA	No	No	Optional	No	No
Kill switch	Multi-modal	No	No	No	No	No
Formal threat model	Yes	Partial	No	No	No	No
Human escalation	Confidence-based	No	Manual	Manual	Manual	Configurable

Enforcement level indicates whether a property is structurally guaranteed (Enforced/Yes/Deterministic), optionally available or developer-configurable (Partial/Configurable/Manual/Optional), or absent (No). Const. AI: Constitutional AI; ECDSA: Elliptic Curve Digital Signature Algorithm. The non-bypassable gate property holds under the stated trusted-infrastructure assumptions (A1–A5, Section 7). This comparison set is the established agent frameworks; newer governance systems are compared in the text and in the quantitative comparison (Table 2).

**Table 2:** Reported performance characteristics of agent governance systems.

System	Latency (p50)	Bypass/Err.	Determ.	Guarantees	Benchmark	Audit
LATTICE (ours)	0.69 ms*^*a*^*	0/21 bypassed*^*f*^*	Deterministic	Cryptographic	130K evals	Hash chain
LGA	980 ms	1.5–6.7%*^*b*^*	Non-det.	Probabilistic	1,081 samples	Immutable logs
AgentSpec	~1 ms*^*c*^*	~9%	Deterministic	Rule-based	3 domains	Rule logs
SAGA	N/A*^*d*^*	0%	Deterministic	Cryptographic	Multiple tasks	Crypto sigs
ArGen	n/a	n/a*^*e*^*	Probabilistic	None	1 domain	Metrics
MS Toolkit	< 0.1 ms	n/a	Deterministic	Rule-based	10/10 OWASP	Policy logs
GaaS	n/a	Trust-dep.	Non-det.	None	2 domains	Trust scores

Values are drawn from each system's own published papers and official releases under heterogeneous evaluation conditions; direct comparison should be interpreted with caution as systems target different domains, use different benchmarks, and report different metrics. (a) Full gated enforcement (signed), including hash verification and audit I/O, measured on the disclosed host (Apple M4 Pro); policy evaluation alone: 6.2 μs. Latency is host-specific. (b) LGA does not report a measured bypass rate; the 1.5–6.7% shown is its reported false-positive rate (best LLM-judge cascade, 93–98.5% interception), shown here for context and not directly comparable to a bypass/error rate. (c) Estimated from “milliseconds” reported in paper. (d) Reported as overhead fraction rather than absolute latency. (e) ArGen modifies model weights at training time rather than enforcing policy at runtime; bypass rate is not directly comparable. (f) Observed in a 21-vector adversarial test suite; see Section 9.9 for confidence bounds.

Constitutional AI scores “No” on structural dimensions because it operates entirely within the model's forward pass; there is no external enforcement layer. AutoGPT and LangChain lack governance layers by design, delegating safety to the developer. CrewAI introduces role separation but does not enforce it architecturally. AutoGen provides configurable human-in-the-loop patterns, earning partial credit for separation of duties and human escalation, but lacks deterministic policy evaluation, non-bypassable gates, and cryptographic audit. Within this comparison set, LATTICE is the only framework that addresses all eight enforcement dimensions simultaneously. The closest architectural peers are SAGA (Syros et al., 2025) and Faramesh (Fatmi, 2026), which match LATTICE on the load-bearing security dimensions (non-bypassability, deterministic verdicts, and cryptographic auditability); within this set LATTICE is distinctive in combining these with tiered confidence-based human escalation, a formal threat model, and a multi-cell supervisory architecture, rather than in any claim of categorical superiority. This field is evolving rapidly; we encourage readers to consult the cited works directly for their most current capabilities.

## 3 Theoretical framework

LATTICE derives its architectural principles from three foundational domains, separation of concerns, functional safety engineering, and regulatory requirements for AI oversight, taking each as a design premise rather than a compliance target.

Separation of concerns (Dijkstra, 1982) and Parnas's complementary principle of information hiding (Parnas, 1972) require that distinct responsibilities, planning what to do, executing actions, and judging compliance, be isolated in components with well-defined interfaces, each hiding its internal state from the others. The decisive consequence is that combining these functions creates a structural conflict of interest: an agent that both decides actions and judges their compliance is biased toward self-approval. LATTICE therefore enforces isolation architecturally, the component that plans actions cannot modify governance rules, and the component that enforces governance cannot execute actions.

Functional safety engineering (IEC, 2010) contributes three requirements that LATTICE adopts directly, safety functions independent from control functions (governance enforcement is independent of agent reasoning), failure to a safe state (failures trigger safe shutdown), and assurance proportioned to risk (confidence thresholds route oversight according to a policy-derived risk signal). Regulatory oversight frameworks supply the remaining constraints: the EU AI Act's requirement of effective human oversight for high-risk systems (European Parliament and Council of the European Union, 2024) and the NIST AI RMF's requirement that governance be testable and auditable (Tabassi, 2023) translate into auditable evidence for every decision, an operator who can always halt operations, and deterministically verifiable governance behavior. Section 2 reviews this literature; here the three domains serve as the premises the architecture is built to satisfy.

### 3.1 From principles to formal assumptions

These three theoretical foundations impose concrete requirements on the architecture that are formalized as assumptions A1–A5 in Section 6. Separation of concerns motivates the isolation assumption (A5), which keeps execution and governance in distinct trust domains. Functional safety's independence requirement motivates the deterministic evaluation assumption (A3) and the append-only audit assumption (A4). Regulatory requirements for verifiability motivate the cryptographic integrity assumptions (A1, A2). Section 7 analyzes the consequences when individual assumptions are violated, so that the framework degrades predictably rather than failing silently.

## 4 Architecture

LATTICE implements governance through a hierarchical grid structure where each Grid Cell contains four functionally isolated components operating under explicit separation of concerns. The architectural design realizes the theoretical principles established in Section 3: Dijkstra's separation of concerns motivates the component isolation, functional safety engineering shapes the fail-closed enforcement model, and regulatory requirements drive the audit and oversight mechanisms.

**Figure 1:**
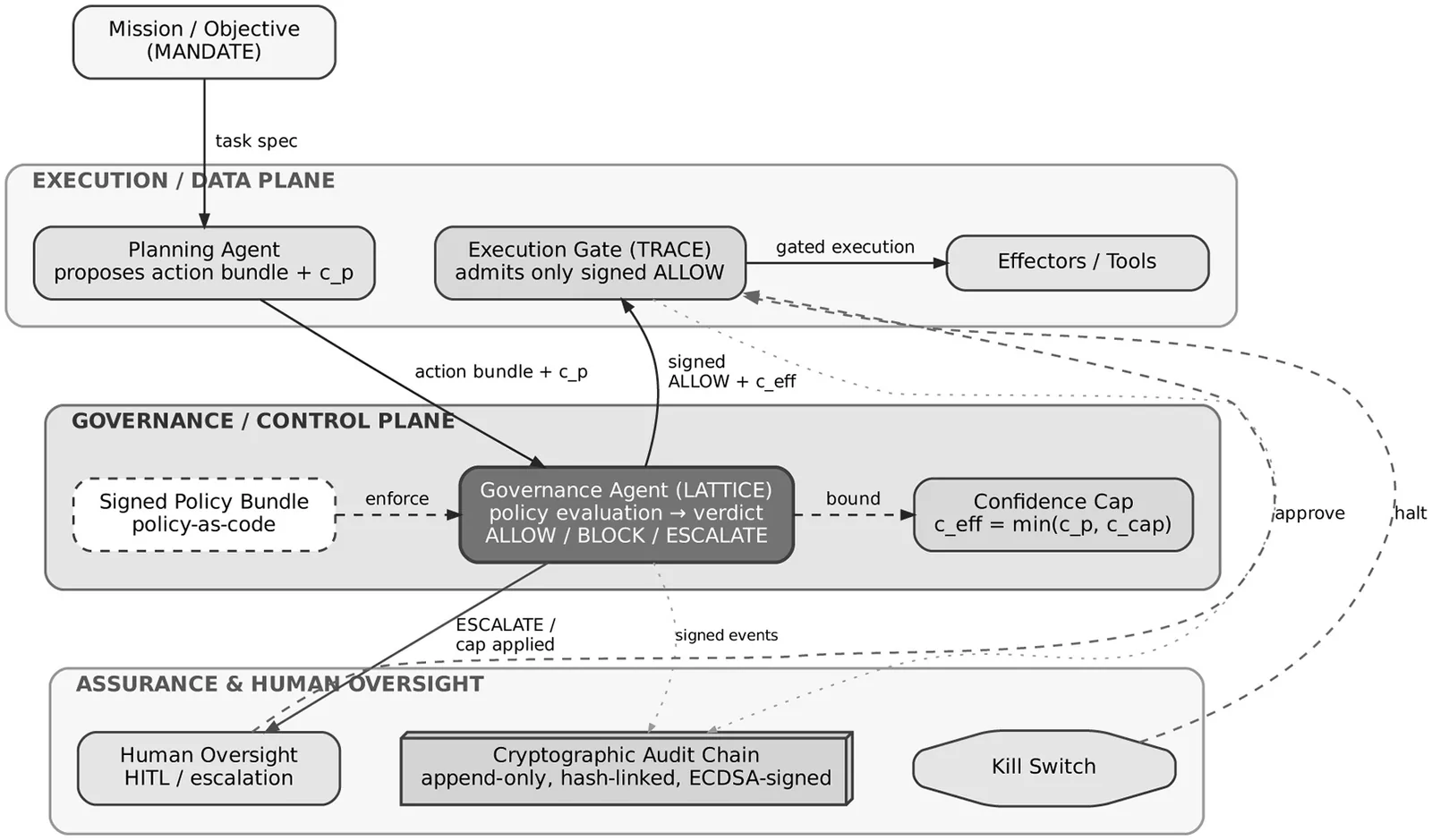
Comprehensive reference model of LATTICE, orienting the detailed specifications that follow. A mission objective (MANDATE) enters the **Execution/Data Plane**, where the Planning Agent proposes an action bundle and a self-reported confidence cp. The **Governance/Control Plane** evaluates the bundle against a signed, content-addressed policy bundle (policy-as-code), producing a deterministic verdict (ALLOW/BLOCK/ESCALATE) and an effective confidence ceff = min(cp, ccap) that a miscalibrated planner cannot inflate (Section 5.6). Only a signed ALLOW reaches the non-bypassable Execution Gate (TRACE) and then the effectors; ESCALATE, or a cap that, when it binds, prevents an autonomous ALLOW, routes the action to **Human Oversight**, and a kill switch can halt execution immediately. (The cap is an architectural mechanism; on the real-planner corpus of Section 9.11 it was an unexercised backstop.) Every component emits signed events to an append-only, hash-linked, ECDSA-signed cryptographic audit chain. The horizontal bands make the plane separation, the architecture's core trust boundary, explicit: planning may use probabilistic models, while governance, gating, and audit are deterministic.

Figure 1 provides a comprehensive reference model of the complete system, spanning the execution/data, governance/control, and assurance planes, as an orientation for the component-level specifications that follow in this and subsequent sections. The remainder of this section details each element: the 1+3 Grid Cell pattern, policy-as-code enforcement, the gated execution model, and the cryptographic audit trail.

### 4.1 The 1+3 grid cell pattern

The fundamental unit of LATTICE organization is the Grid Cell, implementing a 1+3 pattern: one Governance Agent supervising three operational agents (Planning, Execution, and Coordination; Table 3). This pattern enforces separation at the architectural level (no single component can both decide actions and judge compliance).

**Table 3:** Grid cell component responsibilities.

Component	Primary function	Cannot access	Output type
Planning Agent	Decompose objectives into action bundles	Tools, policy modification	Action Bundle (proposed)
Execution Agent	Execute approved actions via tools	Governance rules, direct tool access	Tool results (via gate)
Coordination Agent	Manage inter-cell communication	Local execution, policy evaluation	Coordination messages
Governance Agent	Evaluate policies, issue verdicts	Action planning, tool execution	Verdict (ALLOW/ BLOCK/ ESCALATE)

**Figure 2:**
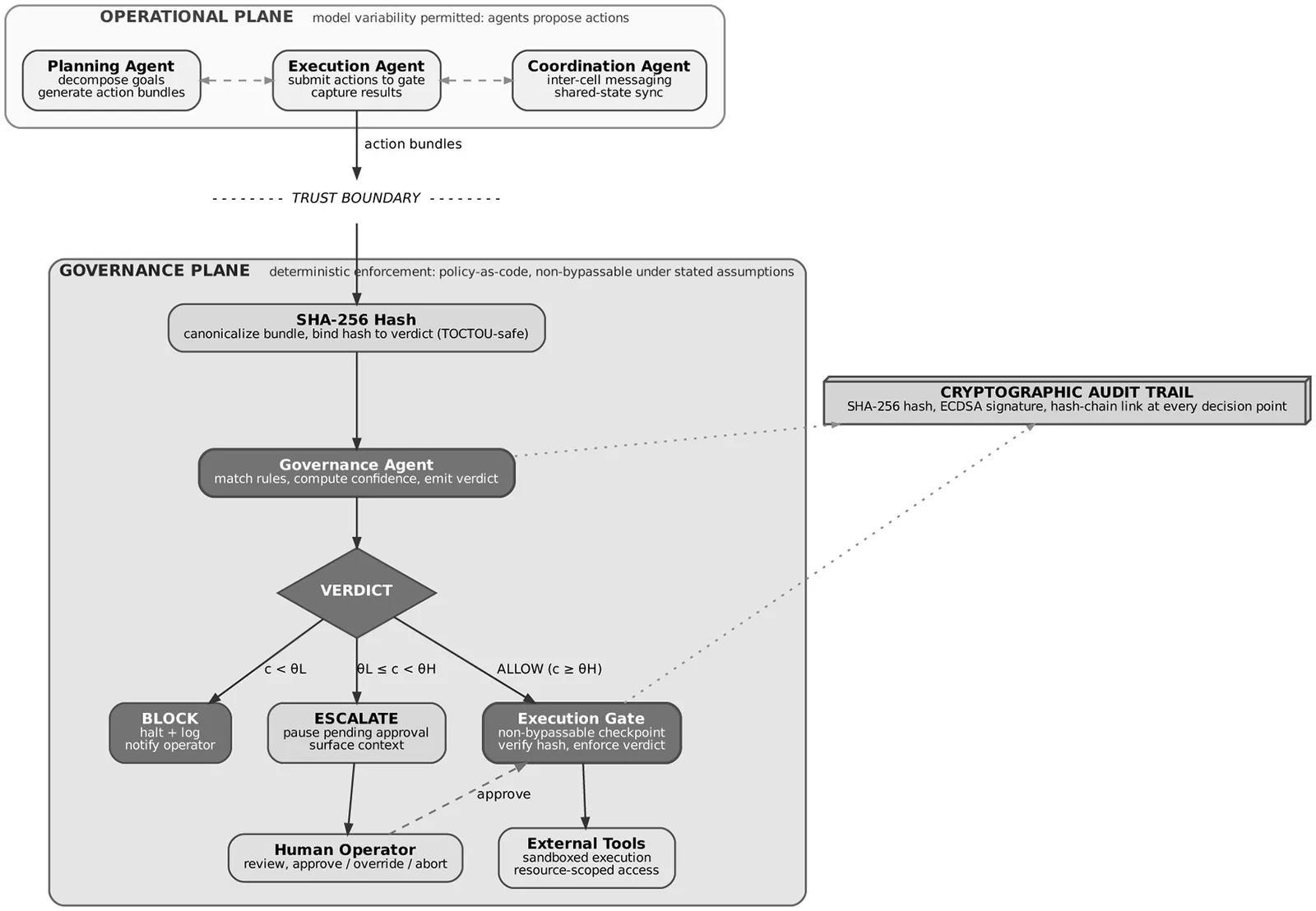
LATTICE trust boundary architecture. The **Operational Plane** (①, top, light fill) permits model variability: three agents, Planning (decomposes objectives), Execution (submits actions), and Coordination (manages inter-cell communication), propose action bundles. The **Governance Plane** (②, bottom, dark fill) enforces deterministic policy: bundles are deep-copied and SHA-256 hashed (③) to prevent TOCTOU mutations; the Governance Agent (④) evaluates against policy-as-code; a deterministic verdict routes to ALLOW (non-bypassable Execution Gate ⑤), BLOCK (⑥, halt with logging), or ESCALATE (human operator review). All steps feed the Cryptographic Audit Trail (⑦).

The 1+3 ratio is not arbitrary. One governance function supervising three operational functions provides adequate oversight capacity while avoiding the overhead of 1:1 supervision. The pattern scales through Grid Cell composition rather than increasing individual cell complexity. Figure 2 illustrates the Grid Cell architecture and the information flow between components. Section 5 specifies the confidence-based decision model that governs action evaluation within each cell, and Section 6 formalizes the safety properties this pattern guarantees.

### 4.2 Policy-as-code enforcement

LATTICE implements policies as deterministic code rather than natural language guidelines. Each policy rule maps action bundle attributes to explicit verdicts: ALLOW, BLOCK, or ESCALATE.

### 4.3 The gated execution model

**Figure 3:**
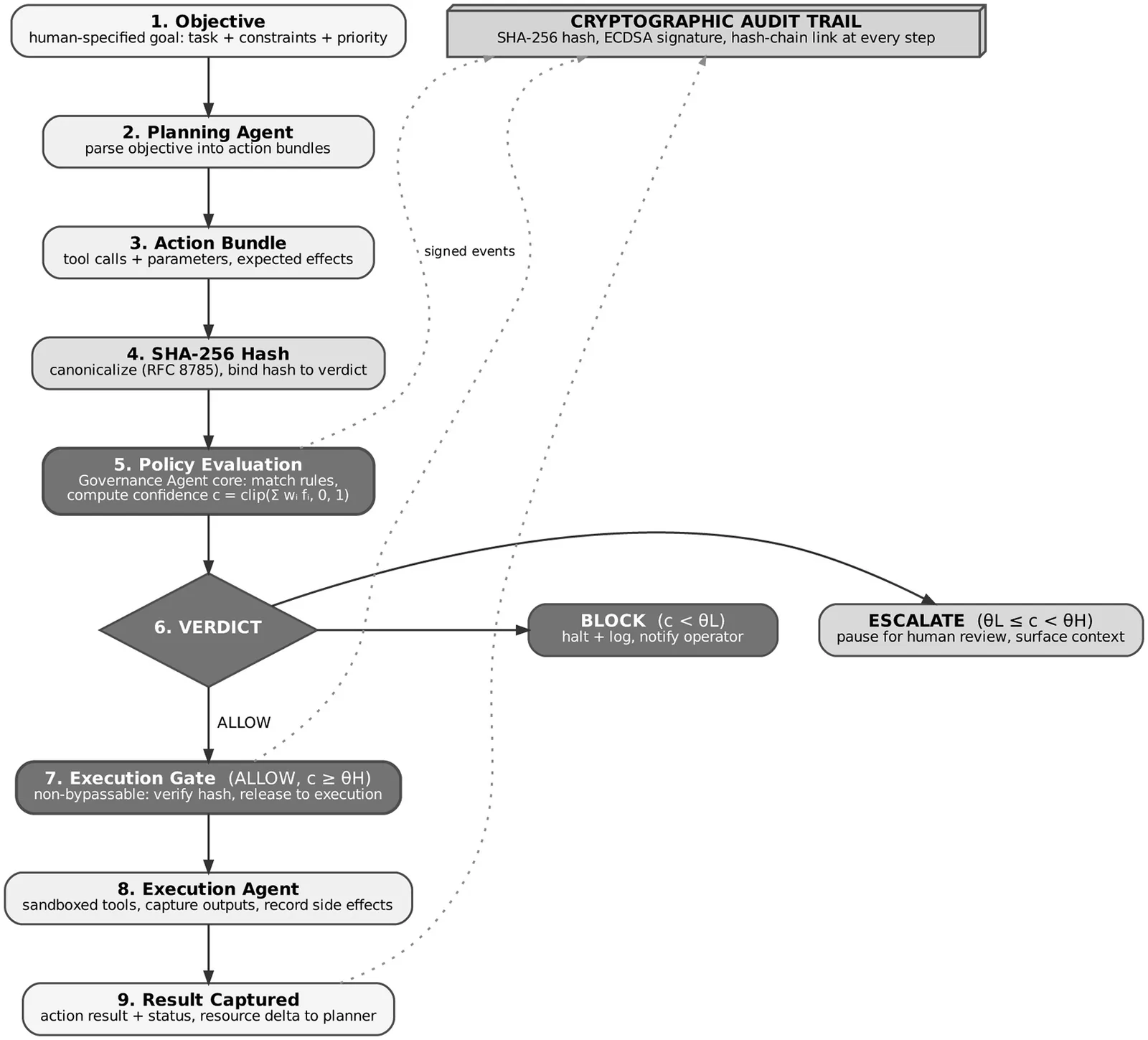
End-to-end governance authorization chain. An objective (①) is decomposed by the Planning Agent (②) into an action bundle (③), which is deep-copied and SHA-256 hashed (④) to prevent TOCTOU mutations. The Governance Agent evaluates the bundle against policy-as-code (⑤) and issues a deterministic verdict (⑥): ALLOW proceeds through the Execution Gate (⑦) for tool access (⑧) and result capture (⑨); BLOCK halts with logging; ESCALATE routes to a human operator. Every step produces a cryptographically signed, hash-chained audit record.

Tool execution occurs only through a Governance-controlled gate that verifies: (1) action bundle hash matches approved bundle, (2) verdict is ALLOW, (3) operation is within time window, and (4) execution context matches approval context. Any verification failure blocks execution and logs the attempt. This gate enforces the Governance Invariance property (Property 4, Section 6.5): no code path to tool execution exists that does not traverse the governance-controlled gate, so that enforcement is structural rather than behavioral. Figure 3 traces the complete authorization chain from objective receipt through policy evaluation, verdict binding, gated execution, and audit capture.

### 4.4 Cryptographic audit trail

Every significant event produces an immutable audit record linked by cryptographic hashes. The chain structure ensures: tamper evidence (any modification breaks the chain), temporal ordering (records cannot be reordered), and attribution (signatures bind records to issuers). These properties are formalized as Evidence Traceability (Property 2, Section 6.3) and validated empirically in Section 9.

## 5 Decision model

LATTICE implements a confidence-based decision model that routes human oversight according to a graduated, policy-derived risk signal. This section specifies the mathematical foundations, component derivation, verdict semantics, threshold calibration rationale, and oversight mode selection.

### 5.1 Confidence scoring

The Planning Agent produces a confidence score *c* ∈ [0, 1] for each proposed action bundle, representing its assessment of action appropriateness given the current objective, context, and available information. The composite score is computed as:


$$\begin{array}{l} c=\text{clip}\left(\overset{5}{\underset{i=1}{\sum }}w_{i}f_{i},\;0,\;1\right) \end{array}$$ (1)

where *f*_*i*_ ∈ [0, 1] represents component factors and *w*_*i*_ represents deployment-calibrated weights satisfying ∑*w*_*i*_ = 1. This score is a governance triage heuristic that routes each action to an oversight level (ALLOW, ESCALATE, or BLOCK); it is not a calibrated estimator of real-world risk. The weights *w*_*i*_ are expert-initialized (Section 5.4), and formal calibration against labeled outcomes is deferred to future work (Section 10.1).

### 5.2 Confidence components

The five component factors are derived from distinct, independently assessable dimensions of action appropriateness:

1. **Objective clarity** (*f*_1_): Specificity of the goal statement. Measures whether the action's purpose is precisely defined (high) or ambiguous (low). Assessed by parsing the action bundle's objective field against the mission specification.
2. **Tool specificity** (*f*_2_): Appropriateness of the selected tool for the task. A tool explicitly listed in the policy bundle for this action class scores higher than a general-purpose tool applied to a specialized task.
3. **Constraint completeness** (*f*_3_): Fraction of required policy constraints that are satisfiable given current context. If the action bundle references a target not yet validated as in-scope, this component decreases.
4. **Risk assessment depth** (*f*_4_): Quality of the risk characterization. Measures whether potential failure modes, side effects, and reversibility have been enumerated and assessed.
5. **Precedent availability** (*f*_5_): Whether similar actions have been successfully executed in prior operations. Novel actions with no precedent score lower, triggering more conservative oversight.

The Governance Agent enforces a fail-closed invariant: if any component *f*_*i*_ is missing or cannot be computed, the engine produces a BLOCK verdict with the reason code CONFIDENCE_COMPONENT_MISSING. This prevents operation with incomplete assessments.

### 5.3 Verdict semantics

The Governance Agent evaluates action bundles against the active policy bundle, producing exactly one of three verdicts:

- **ALLOW:** Action satisfies all policy constraints; permits execution and logs approval.
- **BLOCK:** Action violates one or more constraints; prevents execution and logs violation.
- **ESCALATE:** Action requires human judgment; suspends until operator decision.

The verdict space is intentionally small. Three verdicts suffice because every governance decision reduces to one of three actions: proceed, halt, or defer to a human. Richer verdict spaces (e.g., ALLOW_WITH_MONITORING, CONDITIONAL_ALLOW) can be composed from these primitives combined with policy-level configuration.

### 5.4 Threshold calibration rationale

Default confidence thresholds vary by deployment tier:

- Solo (1 cell): θ_high_ = 0.95, θ_low_ = 0.70
- Team (4 cells): θ_high_ = 0.90, θ_low_ = 0.60
- Squadron (16 cells): θ_high_ = 0.85, θ_low_ = 0.50

The threshold values decrease as deployment scale increases, reflecting two operational realities. First, larger deployments have more operational context and redundancy, so individual actions carry proportionally less risk. Second, human operator bandwidth is a finite resource; at Squadron scale (16 cells), requiring HITL approval above 0.90 would create an unsustainable bottleneck. The thresholds are tunable per deployment and represent conservative defaults informed by human-autonomy teaming research on operator workload management (Parasuraman et al., 2000; Chen and Barnes, 2014). The underlying design goal is *appropriate reliance*: operators should neither over-trust the system (permitting unsafe autonomous actions) nor under-trust it (creating unsustainable escalation volumes). Lee and See (2004) provide a detailed framework showing that trust calibration depends on the interaction between automation characteristics, operator context, and system transparency, precisely the factors that LATTICE's tiered thresholds, auditable verdicts, and confidence component decomposition are designed to support.

Deployments may subdivide the ESCALATE band by introducing additional thresholds within the [θ_low_, θ_high_) interval. For example, the AEGIS reference implementation (Section 9) uses three thresholds, θ_auto_ (corresponding to θ_high_), θ_hitl_ (an internal routing threshold within the ESCALATE band), and θ_esc_ (corresponding to θ_low_), with domain-adapted values (0.85/0.65/0.45) that differ from the architecture defaults to reflect the elevated risk tolerance of offensive security operations. The two-threshold model remains the canonical specification; domain-specific implementations parameterize within this framework.

In practice, threshold and weight calibration follows an iterative process rather than closed-form derivation. The AEGIS reference implementation derived its operational values by starting from the Team-tier architecture defaults, then relaxing thresholds based on domain-specific risk tolerance for offensive security operations. Confidence component weights (*w*_*i*_) were initialized from expert judgment reflecting the relative importance of each factor in the target domain and iteratively refined through tabletop exercises in which operators reviewed governance verdicts on representative action bundles and flagged cases where the verdict conflicted with operational expectations. The threshold sensitivity analysis in Section 9.10 validates that verdict transitions are sharp and predictable across the full confidence range, confirming that the calibrated thresholds produce stable governance behavior. Formal calibration methodologies, including Bayesian optimization of weight vectors against labeled outcome data, remain an open area for future work (Section 10.1).

**Figure 4:**
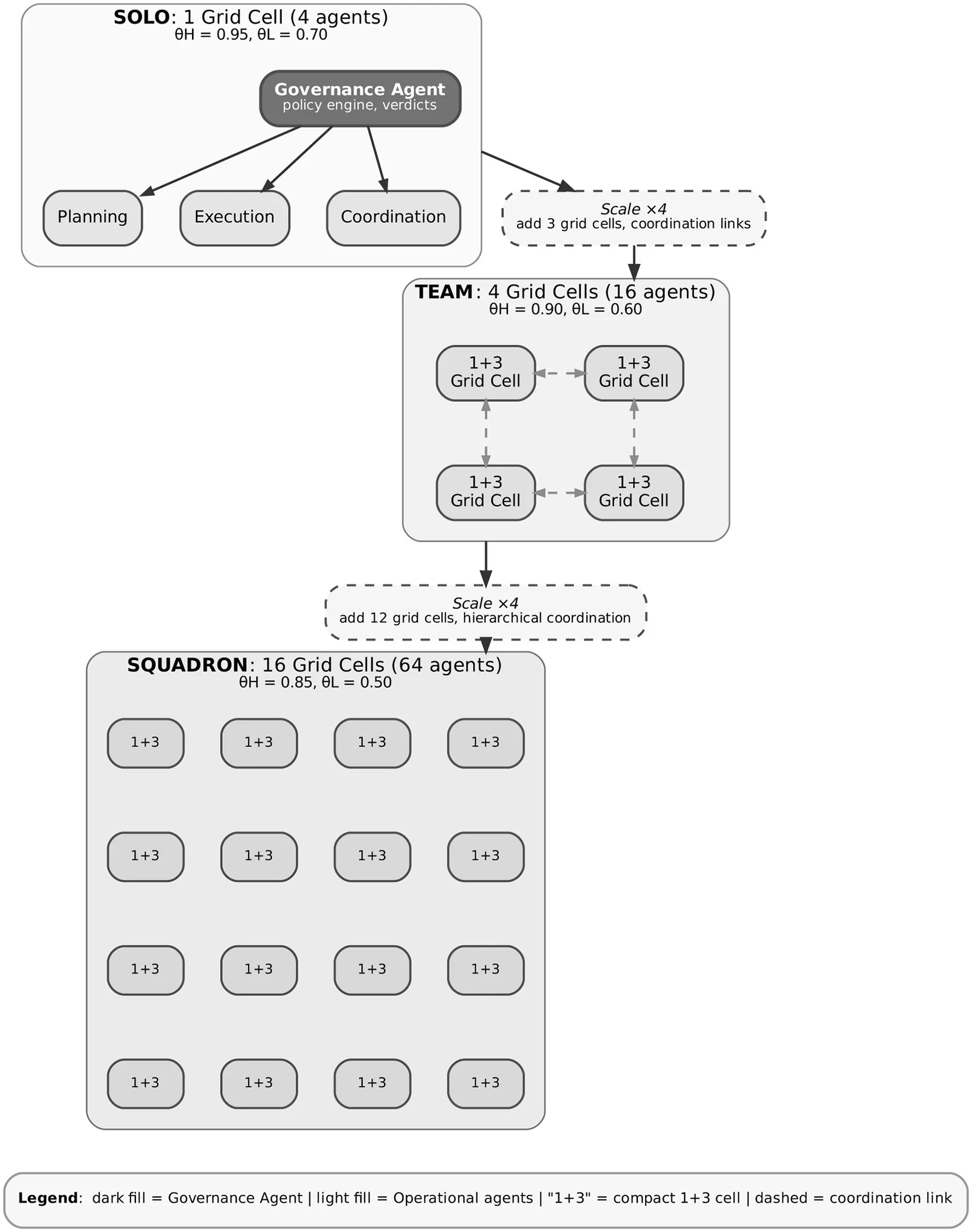
LATTICE deployment scaling tiers. **Solo**: a single 1+3 Grid Cell with conservative thresholds (θH =0.95, θL=0.70) for bounded single-task operations. **Team**: four coordinated Grid Cells (θH =0.90, θL = 0.60); dashed links indicate inter-cell coordination. **Squadron**: sixteen cells in a 4 × 4 mesh (θH = 0.85, θL =0.50) for large-scale campaigns. Thresholds relax at larger scales because greater operational context and redundancy across cells reduce per-action risk; the tiers share no consensus or voting protocol. Each cell maintains independent governance; no cell can override another's policy decisions.

The gap between θ_high_ and θ_low_ defines the HITL band, where human oversight adds the most value. Actions in this range are neither clearly safe nor clearly dangerous. Narrower bands reduce operator interruptions but increase the risk of autonomous execution on borderline actions. Figure 4 illustrates the three deployment tiers and their internal coordination topology.

### 5.5 Oversight mode selection

The combination of confidence score and verdict determines the operational oversight mode (Table 4). Three states guide operations: AUTONOMOUS (action proceeds without human approval), HUMAN-IN-THE-LOOP (action requires explicit operator approval before execution), and HALT (all operations cease and operator is notified).

**Table 4:** Oversight mode determination matrix.

Confidence range	If rule verdict ALLOW	If rule verdict BLOCK	If rule verdict ESCALATE
High (*c* ≥ θ_high_)	Autonomous execute	Autonomous halt	HITL review
Medium (θ_low_ ≤ *c* < θ_high_)	HITL execute	Autonomous halt	HITL review
Low (*c* < θ_low_)	Full stop + notify	Full stop + notify	Full stop + notify

Rows are the confidence tier; columns are the policy-rule verdict.

Each cell is the resulting oversight mode when both conditions hold: the two signals are combined, not redundant. For example, a policy-rule ALLOW with medium confidence yields HITL execution: the rule permits the action but the confidence tier still routes it to a human. Confidence alone never produces an autonomous ALLOW below θ_high_.

Transitions between modes are deterministic given the confidence score and verdict. Emergency stop activation targets < 100ms latency, achieved through a dedicated control channel that bypasses the standard governance evaluation path.

### 5.6 Miscalibration safeguard

LATTICE treats planner-reported confidence as an input to oversight routing, not as an unbounded authorization signal. Because LLMs frequently exhibit systematic overconfidence across diverse tasks (Geng et al., 2024), a planner that consistently inflates its self-assessed score could silently shrink the HITL band and suppress escalations that human operators should review. Two architectural mechanisms mitigate this risk without requiring changes to the underlying model.

#### 5.6.1 Policy-derived confidence cap

Let *c*_*p*_ denote the planner-reported composite confidence (Equation 1) and let *c*_cap_ denote a deterministic ceiling computed by the Governance Agent from policy-observable action attributes: irreversibility class, novelty (no prior precedent in the audit history), privilege level of the requested tool, cross-cell scope, and whether required contingency artifacts (rollback plan, impact assessment) are present. The effective confidence used for verdict routing is


$$\begin{array}{l} c_{\text{eff}}=\min\left(c_{p},c_{\text{cap}}\right). \end{array}$$ (2)

Because *c*_cap_ is derived from governance-observable features, not from the planner's self-report, it cannot be inflated by a miscalibrated or adversarial planning agent. When the cap binds (*c*_cap_ < *c*_*p*_) the engine records CONFIDENCE_CAP_APPLIED and the action is removed from autonomous ALLOW (which requires an empty reason set), routing it to human review regardless of the planner's claimed score. A complementary arithmetic check recomputes the composite from the planner-reported component scores and the policy weights and flags CONFIDENCE_COMPOSITE_DIVERGENCE when the planner's declared composite is inconsistent with it. Both are implemented in the reference engine. The cap is a deterministic backstop against planner over-confidence: in a constructed confidence-inflation stress test, ablating it admits high-consequence actions that the rule layer alone does not catch (high-consequence autonomous ALLOW rate 0.571 with the cap off under an honestly calibrated planner profile, and 0.714 under within-range inflation, both reduced to 0.000 with the cap on; under-reporting is unaffected), whereas in the real-planner safety corpus (Section 9.11) the rule layer and conservative thresholds already intercepted every unsafe action, leaving the cap an unexercised safeguard rather than the binding control.

#### 5.6.2 Statistical audit sampling

Independently, deployments may route a configurable fraction of high-confidence ALLOW decisions to human review on a random-sampling basis. If the sustained operator override rate for a given action class exceeds a configured threshold (indicating that the planner's confidence assessments are systematically uncalibrated for that class), the deployment triggers automatic threshold tightening: θ_high_ for the affected action class is raised until the override rate falls within acceptable bounds. This feedback loop is external to the policy engine and does not compromise determinism; it modifies the *threshold parameters* fed to an otherwise unchanged evaluation function. This sampling loop is a proposed deployment extension; unlike the confidence cap, it is not part of the evaluated reference implementation.

#### 5.6.3 Interaction with existing safeguards

The confidence cap and audit sampling complement rather than replace the fail-closed invariant (Section 5.2) and the independent policy-constraint evaluation (Section 4.2). A missing confidence component still produces a BLOCK verdict, and an out-of-scope or denylisted action is blocked regardless of *c*_eff_. The mechanisms introduced here address the subtler failure mode in which a planner reports *plausible but systematically inflated* scores for action classes that are policy-compliant yet warrant human oversight due to consequence severity.

## 6 Assurance properties

LATTICE provides four assurance properties with explicit assumptions, enforcement mechanisms, and empirically validated verification conditions. Formal property specification for agentic AI is an emerging research direction: Allegrini et al. (2025) define 31 properties in temporal logic (liveness, safety, completeness, fairness) for multi-agent host and task-lifecycle models. LATTICE's properties differ in that they are not merely specified but *architecturally enforced*, each property maps to a structural mechanism that guarantees the property holds by construction rather than requiring runtime verification.

### 6.1 Foundational assumptions

The framework relies on:

- A1: SHA-256 is collision-resistant (NIST FIPS 180-4)
- A2: ECDSA P-256 signatures are unforgeable (NIST FIPS 186-4)
- A3: Policy evaluation engine is deterministic (implementation uses pure functions, no external state)
- A4: Audit store is append-only (implemented via WORM storage or blockchain anchor)
- A5: Sandbox isolation prevents unauthorized access (container/VM isolation with restricted syscalls)

### 6.2 Property 1: governance completeness (safety)

Every action bundle receives exactly one verdict. No action can exist in an unevaluated state, and evaluation always terminates.

Relies on: A3 (deterministic engine)

Enforcement: Finite policy rules; timeout with ESCALATE default; no external dependencies in evaluation path.

### 6.3 Property 2: evidence traceability (safety)

Every executed action has a complete audit chain linking it to its originating objective, policy evaluation, and approval.

Relies on: A1 (SHA-256 collision resistance), A2 (ECDSA unforgeability), A4 (append-only audit).

### 6.4 Property 3: governance determinism (safety)

Given identical action bundle and policy bundle inputs, governance produces the same verdict. This property enables replay verification.

Relies on: A3 (deterministic evaluation engine).

### 6.5 Property 4: governance invariance (safety)

Governance rules remain enforceable regardless of model behavior. This is the core non-bypassability property, which holds *under the stated trusted-infrastructure assumptions* (A1–A5): given those assumptions, the Execution Agent has no code path to tools that does not traverse the Governance-controlled gate. The property instantiates two classical security principles: Anderson's (1972) reference monitor concept, requiring that all resource accesses be mediated by a tamper-proof, always-invoked, verifiable component, and Saltzer and Schroeder's (1975) complete mediation principle, which holds that every access to every object must be checked for authority. Enforcement is structural (the gate is the only interface to tools) rather than behavioral; in the reference implementation this is a *checked* invariant: an automated reachability test confirms the tool-execution primitive is invoked only past an ALLOW verdict, with no other call site in the trusted core. If the sandbox-isolation assumption (A5) fails, the property is not guaranteed; non-bypassability is therefore conditional on the infrastructure rather than unconditional.

Relies on: A5 (sandbox isolation prevents the Execution Agent from accessing tools outside the gate).

Enforcement: Tool APIs are accessible only through the gated interface; the agent's runtime environment has no direct network access, filesystem access, or process control outside the sandbox.

Section 9 presents empirical validation of all four properties against the AEGIS reference implementation.

## 7 Threat model

LATTICE assumes adversarial pressure on the reasoning plane but trusted infrastructure for governance enforcement. This section defines what “trusted infrastructure” means operationally, analyzes failure modes when individual assumptions are violated, and addresses ethical considerations.

### 7.1 Trusted infrastructure definition

“Trusted infrastructure” operationally means: governance components execute in an isolated environment (dedicated VM, container with restricted syscalls, or physical separation); policy stores use encrypted storage with access controls; audit stores are append-only with cryptographic integrity; administrative access requires multi-party authorization; network isolation prevents reasoning-plane components from addressing governance infrastructure.

This definition is deliberately narrow. LATTICE does not assume trust in the LLM, the planning agent's outputs, or the external environment. Christodorescu et al. (2025) formalize this challenge as the “probabilistic Trusted Computing Base (TCB)” problem: in agentic systems, the AI model forms a major component of the TCB yet is fundamentally probabilistic, making classical security invariants unenforceable through the model alone. LATTICE resolves this by excluding the model from the governance TCB entirely: the deterministic policy engine, cryptographic audit chain, and execution gate constitute a *non-probabilistic* TCB that enforces invariants independently of model behavior. The architecture's value lies precisely in constraining untrusted reasoning components through trusted infrastructure. Real-world incidents validate this threat posture: prompt injection attacks have moved from academic research into recurring production incidents, with OWASP ranking prompt injection among the top risks for agentic applications (OWASP Foundation, 2025a). The Microsoft 365 Copilot “EchoLeak” vulnerability (CVE-2025-32711; CVSS v3.1 base score 9.3 assigned by the vendor CNA, 7.5 by the NVD analyst) demonstrated that hidden prompt-injection content in a single crafted email, automatically retrieved into the assistant's context, could silently exfiltrate private organizational data with no user interaction, a concrete instance of the agent goal hijacking that LATTICE's execution gate is designed to prevent (National Vulnerability Database, 2025; Microsoft Security Response Center, 2025; Reddy and Gujral, 2025). Microsoft's April 2026 release of an open-source Agent Governance Toolkit (Microsoft, 2026) further confirms industry convergence on the principle that runtime governance infrastructure, not model-level alignment alone, is necessary for safe autonomous agent deployment.

### 7.2 Partial compromise analysis

A critical question is: what happens when individual infrastructure assumptions fail? We analyze each assumption (A1–A5 from Section 6) independently, identifying the blast radius, residual protections, and detection mechanisms.

#### 7.2.1 A3 violation: policy engine non-determinism

If the policy evaluation engine exhibits non-deterministic behavior (e.g., due to a concurrency bug, floating-point environment difference, or uninitialized state), governance verdicts may vary for identical inputs. **Blast radius**: Replay verification fails; audit records may not reproduce original verdicts. **Residual protections**: The execution gate's TOCTOU check (immutable bundle enforcement via deep-copy isolation) still prevents post-approval mutation. Audit records preserve the actual verdict issued, enabling forensic reconstruction even if replay disagrees. **Detection**: Continuous replay verification in the audit pipeline detects non-determinism within one audit cycle. **Mitigation**: The reference implementation uses pure functions with no external state, mutable global variables, or random number generators. The parametrized test matrix (Section 9.4) validates determinism across 43 input combinations.

#### 7.2.2 A4 violation: audit store compromise

If the append-only audit store is writable by an attacker (e.g., through privilege escalation on the storage host), records could be deleted, modified, or fabricated. **Blast radius**: Evidence traceability (Property 2) is compromised. An attacker could erase records of unauthorized actions. **Residual protections**: ECDSA signatures on individual records remain valid regardless of storage integrity; forging records requires the signing key, which is held separately from the storage system. The hash chain structure means deletion or modification of any record is detectable by any party holding a prior chain checkpoint. **Detection**: External audit anchoring (periodic hash publication to an independent ledger) limits the window of undetectable tampering. **Mitigation**: WORM (Write Once Read Many) storage, geographically distributed replicas, and periodic chain verification reduce the probability and impact of storage compromise.

#### 7.2.3 A5 violation: sandbox escape

If the execution sandbox is breached (e.g., a container escape vulnerability), a compromised agent could directly access governance infrastructure. **Blast radius**: All properties are potentially compromised, as the agent could modify policies, suppress audit records, or invoke tools directly. **Residual protections**: Defense in depth limits cascading failure. The governance agent runs in a separate isolation domain (different VM, not just different container). Policy stores require multi-party authorization for modification, so a single sandbox escape does not grant policy write access. Kill switches include hardware-level mechanisms (network disconnection, power control) that operate independently of software state. **Detection**: Host-level intrusion detection, anomalous network traffic from the sandbox, and governance agent heartbeat monitoring. **Mitigation**: Minimal attack surface in the sandbox (restricted syscalls, no network access to governance infrastructure), regular patching, and nested isolation (VM within a physical security boundary).

#### 7.2.4 Combined failure modes

The most dangerous scenarios involve correlated failures. For example, a sandbox escape (A5) combined with audit compromise (A4) could enable undetected unauthorized actions. LATTICE's defense-in-depth strategy ensures that exploiting this combination requires simultaneous compromise of the sandbox, the audit storage system, the signing key, and any external audit anchors. Each additional layer increases attack cost multiplicatively rather than additively.

#### 7.2.5 Coordination-layer compromise in multi-cell deployments

In Team and Squadron deployments (Section 5.4), the Coordination Agent introduces a system-level threat surface distinct from the per-cell compromise scenarios analyzed in Sections 7.2.1–7.2.4. Because the Coordination Agent exchanges state updates and priority signals across multiple Grid Cells, its compromise can produce *correlated* effects that single-cell governance cannot independently detect. Approaches that translate LLM-generated intent into explicit, tunable coordination policies (de Curtó et al., 2026) offer one route to specifying coordination-layer intent; in the LATTICE model such intent is advisory and is mediated per action by each cell's local governance evaluation, so a compromised or mis-specified coordination policy cannot by itself authorize an action.

##### 7.2.5.1 Blast radius

A compromised Coordination Agent cannot directly open an Execution Gate, modify a cell-local policy bundle, or transfer an ALLOW verdict across cell boundaries; each cell still requires a local governance evaluation before any tool action executes. However, coordination compromise can induce correlated failures through at least four mechanisms:

1. **Message spoofing or replay**. Fabricated or replayed inter-cell messages induce synchronized but incorrect action proposals across multiple cells, each of which may individually appear policy-compliant.
2. **Shared-state poisoning**. Corrupted situational context propagated to multiple Planning Agents causes them to reason from a false operational picture, producing locally valid but globally undesirable action bundles.
3. **Escalation flooding or suppression**. Manipulated inter-cell priority signals either overwhelm operators with spurious escalations [degrading oversight quality through alert fatigue (Tariq et al., 2025)] or suppress legitimate escalations by misrepresenting cross-cell consensus.
4. **Fan-out amplification**. A single malicious coordination event triggers many locally policy-compliant actions that, taken together, produce an effect no individual cell's governance was designed to prevent (e.g., simultaneous reconnaissance against the same target from all cells in a Squadron).

##### 7.2.5.2 Residual protections

The primary risks are correlated misdirection, degraded operator situational awareness, and availability loss across multiple cells, rather than direct bypass of per-cell authorization. Significant residual protections remain: each cell maintains independent governance, no coordination message constitutes an authorization token, and no cell may execute a tool action based solely on another cell's verdict.

##### 7.2.5.3 Mitigations

Coordination-layer integrity requires dedicated architectural controls beyond the single-cell threat model:

- Inter-cell coordination messages should be mutually authenticated, sequence-numbered, time-bounded, and bound to origin-cell identity via correlation IDs recorded in the audit chain.
- Shared-state updates affecting multiple cells should require corroboration from independent sources, quorum among affected cells, or explicit operator approval, graduated by consequence severity.
- Coordination traffic should be treated as untrusted input to local planning rather than as authoritative state; verdict forwarding between cells should be architecturally disallowed.
- Multi-cell actions with coupled effects should require per-cell re-authorization and, for high-consequence action classes, explicit operator approval before coordinated execution proceeds.

##### 7.2.5.4 Scope of the non-bypassability claim

This analysis does not weaken the non-bypassability guarantee at the level of the individual Execution Gate (Section 6.5). It clarifies that coordination compromise is a *separate system-level hazard* that can produce correlated but not directly unauthorized execution, and therefore requires controls dedicated to the coordination plane in addition to the per-cell governance mechanisms validated in Section 9. Empirical validation of multi-cell coordination security, including adversarial testing of the mitigations listed above, is scoped as future work (Section 10.7).

### 7.3 Ethical considerations and dual-use

LATTICE is designed for authorized operations conducted with explicit organizational approval within defined legal boundaries. Because the architecture targets high-consequence settings, including offensive-security operations, where the AEGIS reference implementation deliberately relaxes confidence thresholds relative to the architecture defaults to reflect a different operational risk tolerance, dual-use is central rather than peripheral, and we address it directly.

#### 7.3.1 Authorization: establishment, documentation, revocation

An autonomous deployment is authorized only against an explicit, signed scope. The reference implementation records an *authorization-provenance* entry binding the authorizing principal, the operation, the permitted scope, and an expiry, and maintains a *revocation registry*; an action is admissible only while a matching, unexpired, non-revoked authorization exists. Authorization is thus auditable and revocable, not implicit.

#### 7.3.2 Preventing improper-scope policy bundles

Policy-as-code makes *enforcement* auditable but does not by itself make a policy correct or appropriately scoped. The reference implementation therefore includes a *policy linter* that flags over-broad scope, wildcard target or tool grants, and permissively low thresholds before a bundle is deployed; policy bundles are content-addressed and signed, with versioning and key rotation/revocation, so an unauthorized or tampered bundle is detectable. These mechanisms reduce, though they cannot eliminate, the risk of a bundle encoding a legally or ethically improper scope.

#### 7.3.3 Relaxed thresholds and autonomous-harm risk

Relaxing thresholds widens the autonomous envelope and raises the risk that a high-consequence action proceeds without human review. The policy-derived confidence cap (Section 5.6) is the primary countermeasure: irreversible, high-privilege, cross-cell, or contingency-lacking actions are capped below the autonomous band regardless of the planner's reported confidence, forcing human review even under relaxed thresholds, and hard-safety constraints are enforced as deterministic rules (TIME_WINDOW, PREREQUISITE) rather than via confidence.

#### 7.3.4 Operator accountability

Confidence-based routing can move actions away from human review; to preserve accountability, every verdict, escalation, and execution is recorded in the cryptographic audit chain with attribution, the cap returns high-consequence actions to a named operator, and the authorization-provenance entry binds the operation to an accountable principal. Automation bias and alert fatigue remain real threats to the quality of that review (Section 10.2).

#### 7.3.5 Assessment and release obligations

The governance boundary was exercised by the adversarial suite (Section 9.9) and a pre-specified adversarial safety evaluation (Section 9.11), and the headline statistics were recomputed from the raw artifacts by a separate, author-directed automated pipeline; we regard further independent red-team assessment of the governance boundary as an ongoing obligation, not a one-time check. Because the same architecture that constrains an autonomous agent could lower the operational cost of reconnaissance or exploitation if its governance were misconfigured, captured, or repurposed, responsible release should be accompanied by restriction to authorized, legally-bounded operations, mandatory audit retention, periodic policy and key review, and misuse-prevention review of policy bundles. LATTICE governs structured action attributes; it does not sanitize free-text payloads, which remain an upstream responsibility.

## 8 Compliance mapping

LATTICE does not claim certification to any standard. It provides mechanisms that can support compliance evidence generation.

Key regulatory mappings:

- **EU AI Act**: Addresses human oversight and logging through confidence escalation and audit trails
- **NIST AI RMF**: Supports GOV/MAP/MEASURE/MANAGE through governance lifecycle and monitoring
- **IEC 61508**: Implements safety lifecycle and independence through independent kill switches
- **ISO 13850**: Supports emergency stop principles through multi-modal halt mechanisms
- **ICD 505**: The Intelligence Community Directive on AI governance (Office of the Director of National Intelligence, 2025), implementing the national security AI framework (Office of the President, 2024), mandates risk management, human oversight, and accountability for AI systems; LATTICE addresses these through the Governance Agent, confidence-based escalation, and cryptographic hash chain

### 8.1 OWASP agentic application risk coverage

The OWASP Top 10 for Agentic Applications (OWASP Foundation, 2025a), released in December 2025 by over 100 security researchers, provides the first formal taxonomy of risks specific to autonomous AI agents. Separately, the OWASP Top 10 for LLM Applications (OWASP Foundation, 2025b) identifies Excessive Agency (LLM06) as a top risk, decomposing it into excessive functionality, excessive permissions, and excessive autonomy. Microsoft's March 2026 mapping of Copilot Studio capabilities to the agentic framework (Microsoft Security, 2026) confirms industry-wide adoption of these risk categories as a benchmark for agentic AI security.

Table 5 maps each OWASP agentic risk to the specific LATTICE mechanism that addresses it.

**Table 5:** Mapping of OWASP Top 10 for agentic applications risks to LATTICE mechanisms.

ID	Risk	Description	LATTICE mechanism
ASI01	Agent Goal Hijack	Manipulation of instructions or inputs to redirect agent objectives	Policy-as-code enforcement evaluates actions against declared scope; injected goals produce BLOCK verdicts when resulting actions violate policy constraints
ASI02	Tool Misuse & Exploitation	Agents misuse legitimate tools via prompt manipulation or unsafe chaining	Non-bypassable execution gate requires ALLOW verdict bound to immutable content hash before any tool invocation
ASI03	Identity & Privilege Abuse	Exploitation of delegated trust, inherited credentials, or role chains	Governance Agent evaluates per-action authorization independently; 1+3 Grid Cell isolation prevents privilege inheritance across components
ASI04	Supply Chain Vulnerabilities	Compromised third-party agents, tools, plugins, or update channels	Content-addressed policy bundles with SHA-256 hashing detect tampering; policy version control enables rollback
ASI05	Unexpected Code Execution	Agents generate or execute attacker-controlled code	Execution gate constrains permissible action types; policy rules can explicitly block code execution categories
ASI06	Memory & Context Poisoning	Persistent corruption of agent memory, RAG stores, or context	Architectural separation ensures governance evaluation uses policy state independent of agent memory; poisoned context cannot alter governance verdicts
ASI07	Insecure Inter-Agent Communication	Spoofed or manipulated messages between agents	Cryptographic audit trail with ECDSA signatures provides message authenticity; 1+3 isolation constrains inter-agent communication paths
ASI08	Cascading Failures	False signals propagate through automated pipelines with escalating impact	Confidence-based escalation triggers ESCALATE verdicts at ambiguity boundaries; kill switch halts cascading action chains independently of agent state
ASI09	Human-Agent Trust Exploitation	Polished agent outputs mislead operators into approving harmful actions	Governance verdicts are computed from policy rules, not agent-generated explanations; escalation presents structured action bundles rather than agent narratives
ASI10	Rogue Agents	Misaligned or compromised agents diverge from intended behavior	Non-bypassable gate ensures no action executes without governance approval regardless of agent alignment; multi-modal kill switch provides independent halt

Table 5 is a *conceptual mapping* of LATTICE mechanisms to the ten OWASP agentic risk categories; it is not a claim of exhaustive empirical coverage. The mapping is plausible because governance evaluation is structurally independent from agent reasoning: risks arising from compromised agent behavior (ASI01, ASI06, ASI09, ASI10) are addressed architecturally rather than through behavioral training; tool and identity abuse (ASI02, ASI03, ASI05) are constrained by the gate, non-bypassable under the stated infrastructure assumptions, and per-action policy evaluation; and infrastructure risks (ASI04, ASI07, ASI08) are mitigated by cryptographic integrity and fail-safe escalation. We separately *tested* a representative subset: the 21-vector adversarial suite (Section 9.9) and the real-LLM adversarial families (Section 9.11) exercise scope, tool, time-window, confidence, structural, and coordination attacks, with no in-scope bypass observed. We do not claim every category is exhaustively validated; the mapping identifies where LATTICE's mechanisms apply, and the adversarial evaluations bound how well a representative subset is enforced.

The OWASP LLM Top 10's Excessive Agency risk (LLM06) further validates LATTICE's design. OWASP decomposes excessive agency into three root causes: excessive functionality (agents access tools beyond task scope), excessive permissions (tools operate with broader privileges than necessary), and excessive autonomy (high-impact actions proceed without human oversight) (OWASP Foundation, 2025b). LATTICE addresses all three through scope-constrained policy bundles (functionality), per-action authorization evaluation (permissions), and confidence-based ESCALATE verdicts (autonomy).

## 9 Empirical evaluation

This section presents empirical evidence from the AEGIS reference implementation validating the four assurance properties defined in Section 6. We describe the test methodology, present results across four evaluation dimensions, and report integration validation of the full governance chain. As a roadmap, each sub-study substantiates a specific property: non-bypassability (Section 9.3, Property 4), governance determinism (Section 9.4, Property 3), the confidence/decision model (Section 9.5), audit-chain integrity (Section 9.6, Property 2), end-to-end integration (Section 9.7), latency (Section 9.8), adversarial robustness (Section 9.9), threshold sensitivity (Section 9.10), planner-invariant safety across four LLM families (Section 9.11), and a live closed-loop execution run (Section 9.12); cross-domain transfer is treated separately in Section 10.6.

### 9.1 Reference implementation and test environment

The reference implementation is **AEGIS**, a platform for authorized autonomous offensive-security operations. AEGIS realizes the LATTICE architecture as a Python monorepo of three packages that correspond to the planes of the reference model (Figure 1): **MANDATE** ingests and specifies the mission objective; **LATTICE** performs authorization and governance: the Governance Agent, the policy-as-code engine, the confidence model and cap, and the cryptographic audit chain; and **TRACE** enforces runtime execution control through the non-bypassable execution gate. AEGIS additionally supplies the Planning Agent and the operational context (tooling, targets, and the domain-adapted thresholds discussed below). The governance engine within the LATTICE package is the artifact evaluated in this section: it is released as open source at https://github.com/calboreanu/LATTICE (Apache 2.0) and is byte-identical to the engine AEGIS runs, so the governance results reproduce independently of the proprietary platform. AEGIS itself, its planning agent, operational integrations, and latency-measurement host, is proprietary and is described but not released (Data Availability Statement).

The test environment uses Python 3.11, pytest as the test framework, ECDSA P-256 via the cryptography library for audit signing, and JSON Schema Draft 2020-12 for structural validation. All tests execute deterministically with no external service dependencies.

The governance engine's public test suite comprises 367 passing tests across the policy engine, execution gate, confidence model and cap, cryptographic audit chain, coordination security, and the seven rule types.

**Reproducibility**. All open-tier results reported below, determinism, the threshold sweep, the adversarial suite, the confidence-cap evaluation, and the planner-invariant safety analysis, are regenerated from the public repository by a single documented command; the safety evaluation is pre-specified (its protocol and hypotheses were fixed in the released evaluation package before execution; no external registry was used) and re-runs deterministically on the provided corpus; each reported number is mapped to its producing artifact through a claims-to-evidence index; and the headline statistics were recomputed from the raw artifacts by a separate replication pipeline, run under the author's direction and distinct from the harness that produced the results. Only the absolute latency figures are host-specific (Section 9.8).

### 9.2 Evaluation datasets

The evaluation draws on four datasets, all shipped in the public artifact and constructed entirely from synthetic, placeholder identifiers (e.g., acme.example.com, 10.0.1.0/24), no real targets, operational data, or personal information are used.

**(1) Determinism corpus** (130,000 evaluations). Thirteen governance configurations, spanning policy bundles, threshold settings, and action classes that exercise each verdict path, are each evaluated 10,000 times against fixed action bundles. Its significance is to test *reproducibility*: identical inputs must yield byte-identical verdicts and reason codes, with zero tolerance for non-determinism (Section 9.4).

**(2) Threshold-sweep dataset** (3,003 points). Confidence values sampled across the full [0, 1] range are evaluated under each deployment tier's thresholds. Its significance is to characterize the *decision boundary*: it confirms that verdict transitions are sharp and monotonic rather than erratic, validating the calibration rationale (Section 9.10).

**(3) Adversarial suite** (21 vectors, 7 categories). Hand-constructed attacks, time-of-check/time-of-use mutation, scope evasion, privilege and tool confusion, confidence inflation, malformed-policy injection, and audit tampering, probe the governance boundary directly. Its significance is *security validation*: it measures whether any vector reaches an unauthorized execution (none did; one-sided 95% upper bound 13.3%, Section 9.9), and it surfaced the fail-open defect that motivated the POLICY_RULES_INVALID fix.

**(4) Planner-invariant safety trajectory corpora** (4 × *N* = 1, 000). Agent trajectories generated by four frontier planner families, GPT-5, Claude Sonnet 4.6, Gemini, and Grok-4 (per-planner provenance recorded in the artifact), across multiple operational domains, each trajectory carrying structured action attributes and a ground-truth safety label assigned by the specification-derived rule oracle (Section 9.11). Their significance is to test whether correct *enforcement* translates into *governance* of an autonomous agent's behavior under adversarial conditions, against capability- and threshold-based baselines and self-ablations, and whether that governance is invariant to the planner model. The corpora are shipped so the analysis re-runs deterministically without invoking a model.

### 9.3 Non-bypassability (property 4)

Non-bypassability requires that no action execute without a valid ALLOW verdict from the Governance Agent. We evaluate this property through three categories of tests.

#### 9.3.1 TOCTOU prevention

The execution gate implements immutable bundle enforcement with deep-copy isolation. We validated TOCTOU resistance through adversarial mutation tests where external code mutates the caller's action bundle, policy bundle, and decision tree references *during* policy evaluation. In all cases, the gate operates on its frozen internal copy and returns the correct verdict based on the original inputs. Three specific tests confirm this property:

1. **Action bundle mutation**: An adversary replaces the target from acme.example.com to evil.example.com and the tool from echo to rm_rf during evaluation. The gate returns ALLOW based on the original (valid) bundle. Post-test assertion confirms the caller's bundle was mutated (proving isolation, not ignorance).
2. **Policy mutation**: An adversary replaces allowed_targets with [“*.evil.com”] during evaluation. The gate's audit record retains the original policy ID, confirming it used the frozen copy.
3. **Decision tree mutation**: An adversary modifies the tree's default transition during evaluation. The gate returns ALLOW because its internal copy was never modified.

#### 9.3.2 Structural validation gates

The execution gate validates the complete governance unit (action bundle, decision tree, and tripwire predicates) before proceeding to policy evaluation. A parametrized test matrix covers 10 structural validation cases: valid input, missing tree start node, unknown start node, duplicate signal edges, unknown edge targets, non-list tripwires, non-object predicates, missing predicate metrics, duplicate predicate IDs, and invalid window sizes. All invalid cases produce BLOCK verdicts with specific reason codes (e.g., DECISION_TREE_INVALID:*, TRIPWIRE_PREDICATES_INVALID:*).

#### 9.3.3 Signed audit enforcement

When the require_signed_audit flag is set (production default), the gate blocks execution if no audit signer is provided, returning reason codes SIGNED_AUDIT_REQUIRED and AUDIT_SIGNER_MISSING. This prevents operation in degraded audit configurations.

**Result**: Across all TOCTOU, structural validation, and audit enforcement tests (*N* = 22), the execution gate produced the correct verdict in every case (22/22). No test case achieved unauthorized execution.

### 9.4 Governance determinism (property 3)

Determinism requires identical verdicts for identical inputs. We validate this through a parametrized confidence threshold matrix and a rule effect matrix.

#### 9.4.1 Confidence threshold matrix

The AEGIS reference implementation uses domain-adapted thresholds (θ_auto_ = 0.85, θ_hitl_ = 0.65, θ_esc_ = 0.45), relaxed from the architecture defaults (Table 6: θ_high_ = 0.90, θ_low_ = 0.60 for Team tier) to widen the autonomous operation envelope for offensive security operations, where higher risk tolerance is operationally appropriate. The three AEGIS thresholds map to the two-threshold model as follows: θ_auto_ corresponds to θ_high_ (above which actions proceed autonomously), θ_esc_ corresponds to θ_low_ (below which actions are blocked), and θ_hitl_ subdivides the ESCALATE band for operational routing. Using these thresholds, we evaluated 21 confidence levels spanning the full [0.0, 0.99] range. Results confirm deterministic boundary behavior:

- *c* ≥ 0.85: ALLOW (5 test points: 0.85, 0.88, 0.90, 0.95, 0.99)
- 0.45 ≤ *c* < 0.85: ESCALATE (9 test points: 0.45, 0.50, 0.55, 0.60, 0.65, 0.70, 0.75, 0.80, 0.84)
- *c* < 0.45: BLOCK (7 test points: 0.00, 0.10, 0.20, 0.30, 0.35, 0.40, 0.44)

**Table 6:** Default confidence thresholds by deployment tier.

Tier	θ_high_	θ_low_	Use case
Solo (1 cell)	0.95	0.70	Single bounded task
Team (4 cells)	0.90	0.60	Coordinated operation
Squadron (16 cells)	0.85	0.50	Large-scale campaign

All 21 test points produced the expected verdict with zero variance across repeated executions.

#### 9.4.2 Rule effect matrix

The policy engine supports seven rule types, TARGET_DENYLIST, TOOL_DENYLIST, SCOPE_TAG_DENYLIST, TARGET_ALLOWLIST, TOOL_ALLOWLIST, and the hard-safety types TIME_WINDOW (maintenance/execution-window enforcement) and PREREQUISITE (prerequisite gating), three effect types (BLOCK, ESCALATE, SCORE), and triggered/non-triggered states. A parametrized matrix covering rule type, effect, and trigger-state combinations (plus an unsupported-rule-type edge case) validates that each combination produces the correct verdict deterministically; the two hard-safety types are additionally exercised by dedicated maintenance-window and prerequisite-gating tests. Unsupported rule types produce fail-safe ESCALATE verdicts (RULE_TYPE_UNSUPPORTED:*), and a structurally malformed (non-list) policy fails closed (POLICY_RULES_INVALID) rather than silently skipping enforcement, confirming fail-closed behavior for unknown or malformed inputs.

**Figure 5:**
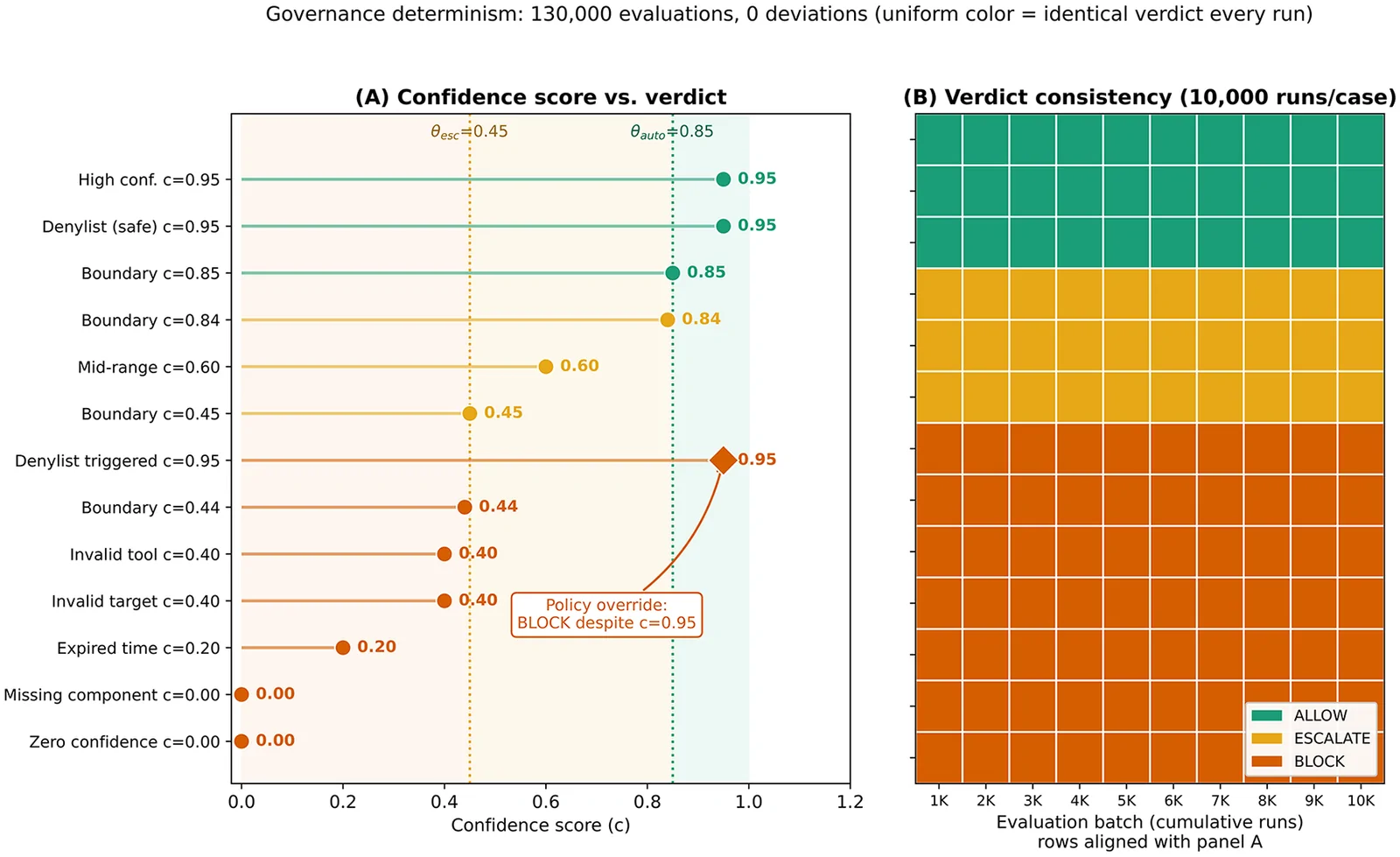
Governance determinism across 13 test configurations. **(A)** Each case plotted at its confidence score, colored by verdict (green = ALLOW, amber = ESCALATE, vermillion = BLOCK). Dashed lines mark thresholds θ_esc_ = 0.45 and θ_auto_ = 0.85. The denylist-triggered case (*c* = 0.95, diamond) demonstrates policy-rule override: BLOCK despite high confidence. **(B)** Consistency matrix: each row shows 10 evaluation batches of 1,000 runs (10,000 total per case, 130,000 overall). Uniform color within every row confirms zero verdict deviation, any inconsistency would appear as a differently colored cell.

**Result**: Across the confidence matrix (*N* = 21) and rule effect matrix (*N* = 22), all 43 test cases produced deterministic verdicts matching specifications with no observed variance. Extended validation across 130,000 evaluations (13 test configurations × 10,000 repetitions) confirmed zero deviations within the tested configuration space. These results demonstrate determinism over the evaluated input domain but do not constitute proof of determinism across all possible inputs. Figure 5 presents the full determinism heatmap.

### 9.5 Confidence model validation (decision model)

The confidence scoring model computes a composite score from the five components defined in Section 5.2. We validate the model's boundary behavior and fail-closed properties.

#### 9.5.1 Five-component scoring

Each component contributes to the composite score on a normalized [0, 1] scale. Tests confirm that:

- High composite scores (*c* = 0.95) produce ALLOW verdicts with confidence mapped to the percentage scale (95.0).
- Mid-range scores (*c* = 0.60) produce ESCALATE verdicts.
- Low scores (*c* = 0.20) produce BLOCK verdicts with the reason code CONFIDENCE_TIER_BLOCK.

#### 9.5.2 Fail-closed on missing components

When any required confidence component is absent (e.g., objective_clarity removed from the score object), the engine produces a BLOCK verdict with the reason code CONFIDENCE_ COMPONENT_ MISSING: objective_ clarity. This prevents operation with incomplete confidence assessments.

#### 9.5.3 Threshold normalization

The engine accepts thresholds on both percentage (85, 65, 45) and normalized (0.85, 0.65, 0.45) scales, correctly mapping confidence scores to verdicts in both representations. This ensures deployment flexibility without ambiguity.

**Result**: The confidence model produces correct verdicts across all tested boundary conditions (*N* = 5 dedicated tests) and exhibits fail-closed behavior on incomplete inputs.

### 9.6 Audit chain integrity (property 2)

Evidence traceability requires that every executed action has a complete, tamper-evident audit chain. We validate creation, sealing, tamper detection, key verification, and immutability properties.

#### 9.6.1 Signed chain round-trip

An ECDSA P-256 keypair is generated, two audit records are appended (a governance verdict and a TOCTOU check), and the chain is sealed with reason OPERATION_COMPLETE. The chain is then loaded and verified using the public key. Verification confirms: all signatures are valid, all hash links are intact, and the seal record contains the correct total record count.

#### 9.6.2 Tamper detection

After creating a valid signed chain, the confidence value in the second record is modified from its original value to 12.34. Verification detects the tampering and reports a “hash mismatch” error. The same test confirms that modifying any field in any record breaks the chain.

#### 9.6.3 Wrong key rejection

A chain signed with key *K*_1_ is verified against a different public key *K*_2_. Verification correctly rejects the chain with a “signature invalid” error. This confirms that audit records cannot be forged without access to the signing key.

#### 9.6.4 Post-seal immutability

After sealing a chain, any attempt to append additional records raises a ValueError with the message “immutable.” This prevents post-hoc record injection.

#### 9.6.5 Unsigned record rejection

When signature requirement is enabled, attempting to append an unsigned record raises a ValueError. This ensures the chain cannot degrade to an unsigned state.

**Result**: All five audit chain properties (signed round-trip, tamper detection, key rejection, post-seal immutability, unsigned rejection) pass with zero failures (N=5 tests).

### 9.7 Integration validation

Beyond unit-level property verification, we validated the complete MANDATE → LATTICE → TRACE governance chain through a 36-step integration scenario (the “Normal Mission” scenario). This scenario exercises specification generation, authorization, and runtime execution control in sequence.

#### 9.7.1 Scenario design

The Normal Mission defines three courses of action (Conservative, Moderate, Aggressive) with 12-node task DAGs, tool bindings across three classes (RECON, SCAN, EXPLOIT), scope constraints (10.0.1.0/24 and acme.example.com), a 4-hour execution window, explicit prohibitions (data exfiltration, destructive actions), and 14 tripwire predicates for runtime monitoring.

The 36 steps map to the three governance phases: Steps 1–8 exercise MANDATE specification and validation, Steps 9–13 exercise LATTICE authorization and bundle signing, and Steps 14–36 exercise TRACE runtime execution with evidence chain construction and verification.

#### 9.7.2 Integration debugging

Initial integration revealed six errors across framework boundaries, each resolved through targeted fixes:

1. Dependency version mismatch (jsonschema < 4.x lacked Draft202012Validator)
2. Module import misattribution across framework boundaries
3. Rug-pull hash computation mismatch between bundle registration and runtime verification
4. Float normalization across LATTICE-TRACE boundary (RFC 8785 JCS strictness)
5. Evidence chain file accumulation across test runs (append-mode persistence)
6. Sandbox permission constraints (file deletion vs. truncation)

A subsequent validation on a different platform (macOS) revealed 27 additional schema validation issues across three categories: float values where string enums were required, missing required fields in trace entry objects, and constraint grammar violations in off-nominal triggers. All were resolved, producing a clean validation: MANDATE valid: True (0 issues), LATTICE verdict: ALLOW, TRACE success: True.

#### 9.7.3 Cryptographic chain of custody

The integration validates an end-to-end hash chain: anchor_hash (MANDATE) → mandate_hash → bundle_hash (LATTICE) → evidence_chain_genesis (TRACE) → chain seal verification. Tampering at any layer breaks downstream verification. The evidence chain uses ECDSA P-256 signatures, and the final seal record contains a cryptographic digest of the complete chain.

**Result**: All 36 integration steps pass. The governance chain produces a cryptographically verifiable evidence trail spanning specification, authorization, and execution.

### 9.8 Performance benchmarks

**Figure 6:**
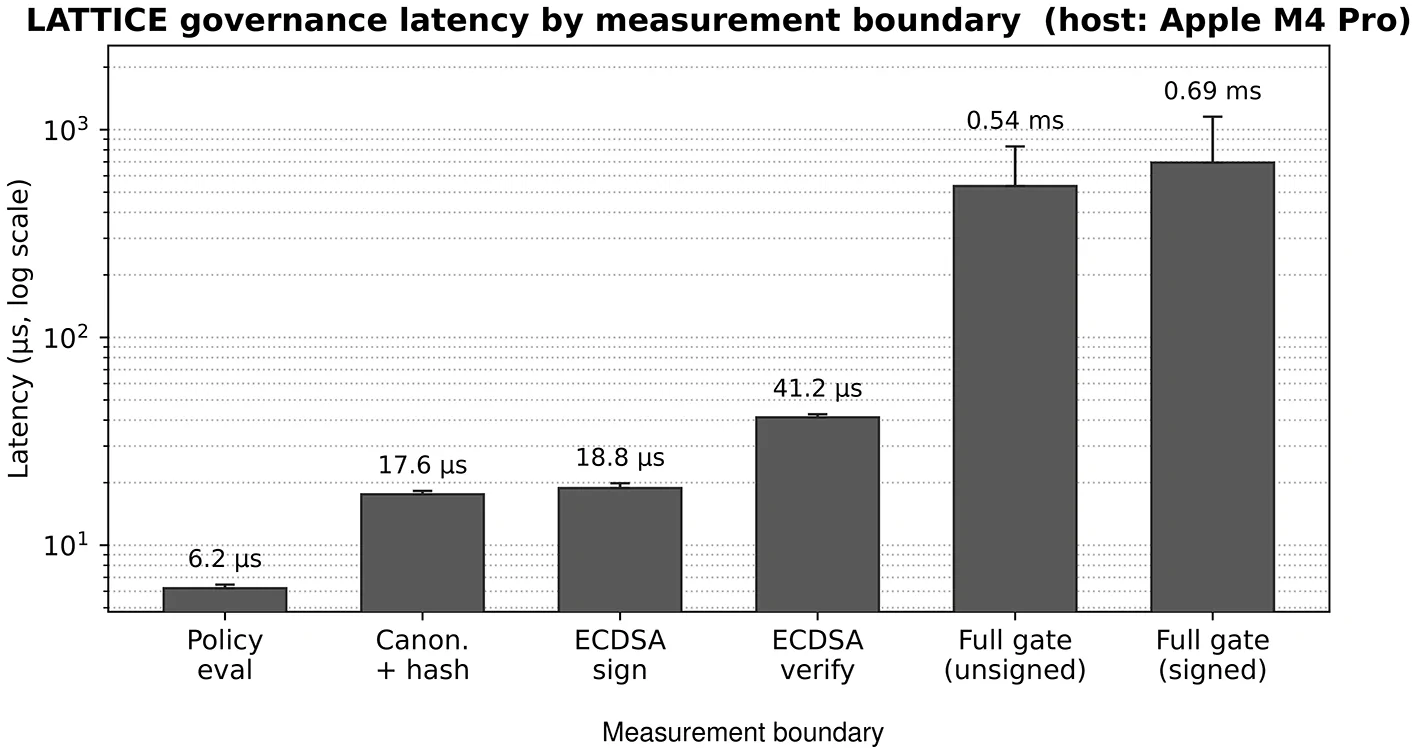
Latency of LATTICE governance operations by measurement boundary, measured on the disclosed host (Apple M4 Pro); bars show p50 with p95 whiskers on a log scale. Policy evaluation P50 = 6.2 μs; canonicalization plus hashing 17.6 μs; ECDSA sign/verify 18.8/41.3 μs; full gated enforcement including audit I/O 0.54 ms (unsigned) and 0.69 ms (signed). Policy evaluation is *O*(*n*) in the number of policy rules. Latency is Tier-2 (host-specific) and varies by host.

We measured latency overhead introduced by the governance evaluation path. All measurements use time.perf_counter_ns() with warmup iterations excluded. Latency is implementation- and host-specific (Tier 2): the values below were measured on an Apple M4 Pro (macOS, Python 3.14; the functional test environment above uses Python 3.11, as latency is host- and runtime-specific and reported separately), and we report each against its explicit measurement boundary, policy evaluation only, canonicalization plus hashing, cryptographic signing/verification, or full gated enforcement including audit I/O, so that boundaries are not conflated across systems. Figure 6 presents the profile.

#### 9.8.1 Policy evaluation latency

Over 5,000 iterations on the disclosed host, policy evaluation alone (the deterministic verdict computation) was: p50 = 6.2 μs, p95 = 6.5 μs, mean = 6.3 μs. Canonicalization plus SHA-256 hashing of the governed unit adds p50 = 17.6 μs. Policy evaluation is *O*(*n*) in the number of policy rules.

#### 9.8.2 Full gate enforce latency

The complete enforcement path (deep-copy inputs, canonicalize, hash, evaluate, write audit record, rehash for the TOCTOU check) over 1,000 iterations: p50 = 0.54 ms (p95 = 0.83 ms) without audit signing, and p50 = 0.69 ms (p95 = 1.15 ms) with ECDSA-signed audit records. The dominant cost is audit-record persistence (file append with JSON serialization); production deployments using append-only databases rather than JSONL files would reduce it substantially.

#### 9.8.3 Cryptographic operations

ECDSA P-256 operations over 2,500 iterations: signing p50 = 18.8 μs (p95 = 19.9 μs) and verification p50 = 41.3 μs (p95 = 42.7 μs). A signature is produced for each audit record and verified on replay.

### 9.9 Adversarial bypass testing

We developed a 21-vector adversarial test suite targeting seven attack categories: scope violations (out-of-scope target, wildcard injection, empty target, empty allowlist), tool violations (unpermitted tool, tool class escalation, empty tool list), time window attacks (expired, malformed, empty timestamps), confidence manipulation (inflated >1.0, negative, missing components, non-numeric types, list-typed values), structural attacks (malformed decision trees, dangling edges, duplicate tripwire IDs), TOCTOU mutation via concurrent threads, and audit/policy violations (missing signer, type confusion).

**Figure 7:**
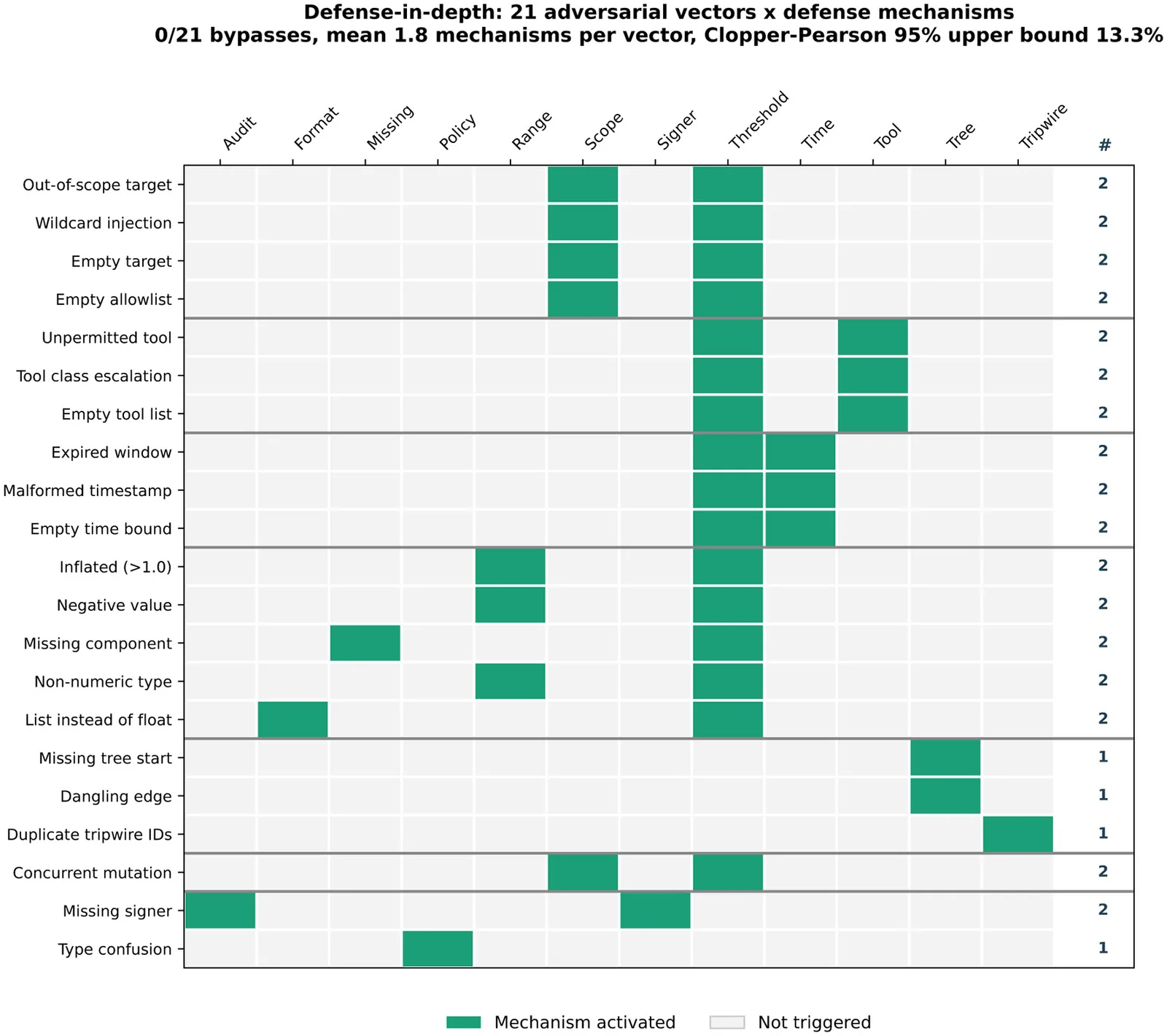
Defense-in-depth analysis of 21 adversarial attack vectors across seven categories. The heatmap shows which defense mechanisms (columns) activated for each attack vector (rows). Most attacks trigger multiple independent mechanisms (mean 1.8 per vector), demonstrating layered defense. Categories: scope violations (4 vectors), tool violations (3), time window attacks (3), confidence manipulation (5), structural attacks (3), TOCTOU mutation (1), and audit/policy violations (2). No bypass was observed (0/21); the one-sided Clopper–Pearson 95% upper bound on bypass probability is 13.3% (*k* = 0, *n* = 21).

Figure 7 presents the results by attack category.

**Result**: All 21 adversarial vectors in the evaluated suite were blocked (0/21 bypassed). Every attack produced either a BLOCK verdict or was handled gracefully. With *n* = 21 and *k* = 0 successes, the exact one-sided 95% confidence upper bound on the true bypass probability is 1−(0.05)^1/21^ ≈ 13.3%; a larger attack surface evaluation is needed to establish tighter bounds. Specific findings include: inflated confidence scores (>1.0) are caught by range validation (CONFIDENCE_COMPONENT_OUT_OF_RANGE), concurrent bundle mutation during evaluation is defeated by the deep-copy isolation, and non-numeric confidence values produce CONFIDENCE_COMPONENT_INVALID reason codes.

### 9.10 Threshold sensitivity analysis

**Figure 8:**
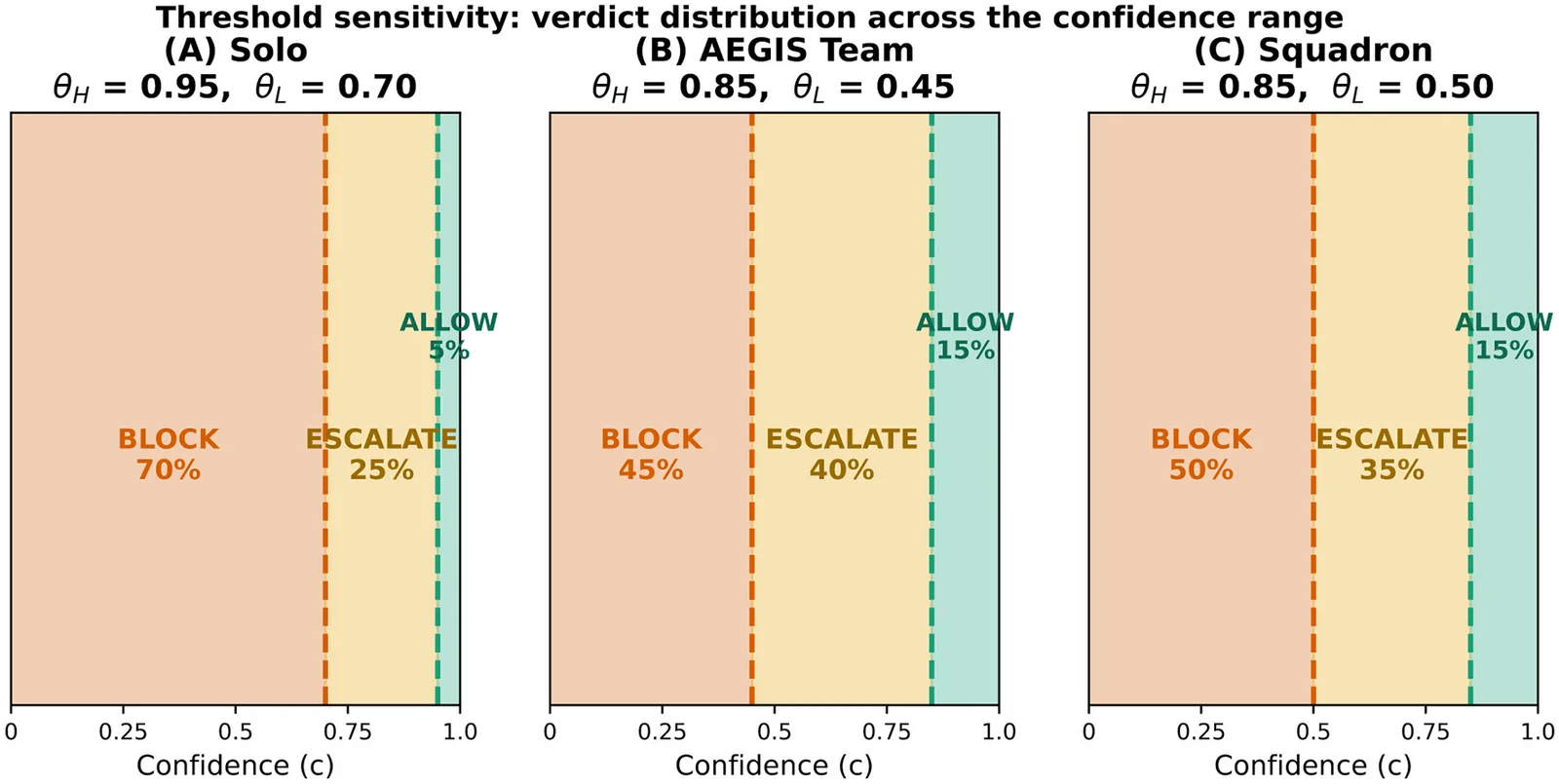
Threshold sensitivity analysis as stacked area charts showing verdict distribution across the full confidence range [0, 1] for each deployment tier. **(A)** Solo tier (θ_*H*_ = 0.95, θ_*L*_ = 0.70): most restrictive, with 70% BLOCK, 25% ESCALATE, and only 5% ALLOW. **(B)** AEGIS Team tier (θ_*H*_ = 0.85, θ_*L*_ = 0.45): balanced profile with 45% BLOCK, 40% ESCALATE, 15% ALLOW. **(C)** Squadron tier (θ_*H*_ = 0.85, θ_*L*_ = 0.50): widest BLOCK region at 50%, with 35% ESCALATE and 15% ALLOW. Sharp transitions at all threshold boundaries confirm deterministic verdict assignment with no fuzzy boundary behavior.

We swept confidence scores from 0.0 to 1.0 at 0.001 resolution (1,001 evaluation points per tier) to characterize how verdict distributions change across deployment tiers and threshold settings. Figure 8 visualizes the verdict transitions across all three tiers.

For the AEGIS Team configuration (domain-adapted θ_auto_ = 0.85, θ_esc_ = 0.45; the architecture-default Team tier is 0.90/0.60, Table 6): 15.1% of the confidence space maps to ALLOW, 40.0% to ESCALATE, and 45.0% to BLOCK (boundary-inclusive fractions of the 1,001-point grid; the analytic fractions are 15.0/40.0/45.0). Boundary analysis at 0.0001 resolution confirms sharp transitions: the ESCALATE → ALLOW boundary occurs precisely at *c* = 0.85, and the BLOCK → ESCALATE boundary at *c* = 0.45. No fuzzy regions or non-deterministic behavior exist near thresholds.

Threshold variation analysis (fixing θ_esc_ = 0.45, sweeping θ_auto_ from 0.70 to 0.99) shows that the ALLOW region shrinks linearly: from 30.1% at θ_auto_ = 0.70 to 1.1% at θ_auto_ = 0.99. This confirms that threshold tuning provides predictable, continuous control over the autonomous operation envelope.

### 9.11 Planner-invariant safety evaluation (real-LLM)

The property tests above establish that the AEGIS reference implementation enforces its policy correctly and deterministically. To assess whether that enforcement *governs* an autonomous agent's behavior, we ran a pre-specified, planner-invariant safety evaluation across **four frontier planner families**, OpenAI GPT-5, Anthropic Claude Sonnet 4.6, Google Gemini, and xAI Grok-4, each generating *N* = 1, 000 agent trajectories (4,000 total) across operational domains, with each planner's trajectories independently labeled by an LLM judge from a *different* model family (GPT-5 judged the Claude and Gemini corpora; Claude Sonnet 4.6 judged the GPT-5 and Grok-4 corpora), an application of the cross-model independent-audit method established in companion work (Calboreanu, 2026a). Ground-truth safety labels are assigned by a transparent, specification-derived rule oracle independent of the engine; hypotheses were pre-specified with multiplicity control (Holm), and the headline figures were recomputed from the raw corpus by a separate replication pipeline, run under the author's direction and distinct from the harness that generated the results.

#### 9.11.1 Planner-invariant safety (H1, H8)

Across all four planner families, the AEGIS reference implementation permitted **zero** unsafe actions: false-allow rate **0.0** with recall 1.0: every oracle-flagged action was escalated or blocked. The same metric was 1.0 for an ungoverned baseline (all planners) and ranged from **0.03 to 0.998** for a confidence-threshold baseline *depending on the planner model*. Governance enforcement is therefore invariant to the planner (H8: maximum false-allow 0.0, spread 0.0 across families), whereas the baseline's safety depends strongly on which model plans. This invariance claim is confined to the operational false-allow metric; the held-out adversarial split is engine-level (identical across planners), with in-scope structured-attribute bypass 0/142 and an overall held-out bypass of 19.3% (34/176) arising entirely from the out-of-model free-text injection family that an upstream input-sanitization layer is responsible for (Section 10.1), and the recall of 1.0 is attained at a conservative, escalation-heavy operating point (no action was auto-allowed), so it should not be read as evidence of selective accuracy.

#### 9.11.2 Autonomy is not assessed by this run

The AEGIS reference implementation auto-approved *none* of the actions, escalating or blocking all of them for every planner. The cause is an artifact of the evaluation's policy encoding rather than planner confidence: autonomous ALLOW required a specific *structured* prerequisite token (an explicit isolation-verified flag), which the planners supplied only as free-text prerequisite descriptions, so nearly every action was escalated for an unmet structured prerequisite, independent of self-reported confidence (one planner's median composite confidence was 0.94, well above the 0.85 bar, yet its actions were still escalated). This run therefore evidences planner-invariant *safety*, no unsafe action was admitted, but is uninformative about autonomous *throughput*, which would require a policy whose prerequisite encoding matches the planner's output schema (Section 10.1). The live closed-loop run in Section 9.12 partially addresses autonomy, exercising the authorize-and-execute path that this operating point did not.

#### 9.11.3 Labeling reliability

The independent, different-family LLM judges agreed only weakly with the specification oracle (Cohen κ from −0.05 to 0.28; agreement 0.24–0.46): the judges were markedly more lenient, rarely issuing a BLOCK. We therefore treat the transparent rule oracle, not an LLM judge, as ground truth and report LLM-judge agreement only as a (low) labeling-reliability bound. The divergence is itself a finding: a contemporary LLM is an unreliable adjudicator of a strict operational safety specification, which is precisely why LATTICE's ground truth is a transparent rule oracle rather than a model.

#### 9.11.4 Scope and validity

The result is correct, planner-invariant prevention of unsafe *structured* actions under a transparent oracle. The separation from the confidence-threshold baseline (0.0 vs. 0.03–0.998) is a descriptive full-set contrast; in the pre-specified confirmatory test (McNemar with Holm correction; Supplementary Table S17) the reference implementation separates from the *ungoverned* baseline for three of four planners, while against the confidence-threshold baseline the paired test is uninformative, since both admit zero unsafe actions on the judge-agreed subset. Clopper–Pearson intervals and bootstrap resampling accompany the reported rates. It is not a field-efficacy claim: the oracle shares LATTICE's structured-attribute ontology, autonomy was low at the configured thresholds, the independent-judge agreement was weak, and there was no live human-subject deployment (Section 10.1). The policy-derived confidence cap was *not separately exercised* by this corpus, the rule layer and conservative thresholds already intercepted every unsafe action, so ablating the cap changed no verdict; its value is shown instead as a targeted safeguard in Section 5.6. The four-planner corpus, the multi-model harness, and the cross-model summary are released with the evaluation package.

### 9.12 Live closed-loop execution

The results above evaluate the governance *decision*. To close the loop, the released engine also governed *real, side-effecting* operating-system actions: each action passed through the execution gate (execution_gate.enforce); an ALLOW verdict invoked a confined real effector (filesystem and process operations restricted to a workspace with a fixed command allowlist), while ESCALATE or BLOCK withheld it, and the on-disk result was then verified independently. The effector is a public stand-in for the execution seam used by the AEGIS reference implementation; it and the engine both ship in the evaluation package and re-run with a single command, so the loop is reproducible rather than a closed demonstration.

Across eight representative actions the gate auto-allowed and executed the three in-scope operations (an in-scope write, a directory creation, and a read-only listing) and withheld the five out-of-scope, missing-prerequisite, irreversible-without-contingency, or low-confidence operations; every on-disk effect matched the expected outcome (Supplementary Table S28). A 10,000-action burn-in sustained this behavior: 6,000 actions were auto-allowed and executed, 4,000 were escalated or blocked, with **zero** unsafe executions, a verifying ECDSA audit chain, bounded latency drift (1.13×), and 1.1 MB resident-memory growth (Supplementary Table S29).

This run exercises the autonomous ALLOW-and-execute path that the four-planner operating point (Section 9.11) did not: there an unmet structured prerequisite escalated every action, whereas here, where the actions can meet the policy's prerequisites, the gate authorizes and executes the safe actions and withholds the unsafe ones, with no unsafe execution. This establishes that the conservative four-planner result reflects that run's prerequisite encoding rather than an inability of the architecture to authorize autonomous action. The run is reproducible reference-implementation validation on the open engine, with a constructed action set and policy-derived expected outcomes; it demonstrates correct, safe authorize-and-execute behavior in a real closed loop and complements, rather than substitutes for, an independent field-efficacy study at scale.

### 9.13 Summary of empirical results

Table 7 summarizes the empirical evaluation across all dimensions.

**Table 7:** Summary of empirical evaluation results.

Property	Tests/evals	Pass	Key finding
Non-bypassability	22 tests	22/22 pass	No unauthorized executions observed across TOCTOU, structural, and audit enforcement tests
Determinism	130,000 evals	0 deviations	Zero variance observed across 13 test cases × 10,000 repetitions
Confidence model	5 tests	5/5 pass	Fail-closed on missing components; correct tier mapping
Audit chain	5 tests	5/5 pass	Tamper detection, key rejection, post-seal immutability
Integration chain	36 steps	36/36 pass	End-to-end hash chain across MANDATE → LATTICE → TRACE
Adversarial bypass	21 vectors	0/21 bypassed	All 7 tested attack categories blocked
Planner-invariant safety (real-LLM, 4 planners)	4 × 1,000	n/a	False-allow 0.0, recall 1.0, planner-invariant; threshold baseline 0.03–0.998
Live closed-loop exec.	8 + 10,000	0 unsafe	Auto-allows and executes in-scope actions, blocks the rest; 6,000 executed in soak, 0 unsafe
Latency (policy eval)	5,000 iters	n/a	p50 = 6.2 μs, p95 = 6.5 μs (host-disclosed)
Latency (full gate)	1,000 iters	n/a	p50 = 0.69 ms signed / 0.54 ms unsigned
Sensitivity	3,003 points	n/a	Sharp threshold transitions; linear control over autonomy envelope

### 9.14 Threats to validity

#### 9.14.1 Internal validity

Tests execute against the reference implementation, not a production deployment. The policy engine uses pure functions with no external state, reducing (but not eliminating) the risk of environment-dependent behavior.

#### 9.14.2 External validity

The evaluation covers one reference implementation (AEGIS) in one domain (autonomous security operations). Generalization to other domains (medical, financial, critical infrastructure) requires additional validation with domain-specific policy bundles and threat models.

#### 9.14.3 Construct validity

Non-bypassability is validated through known attack vectors (TOCTOU mutation, structural manipulation, audit degradation). Sophisticated attacks not yet conceived could reveal vulnerabilities not covered by the current test suite.

#### 9.14.4 Statistical power

The confidence threshold matrix tests 21 discrete points across the [0, 1] range; the separate sensitivity sweep (Figure 8) covers the same range at 0.001 resolution with 0.0001-resolution boundary confirmation, mitigating boundary-behavior concerns. The remaining statistical limitations concern the representativeness of the evaluated confidence distributions and malformed-confidence encodings beyond those exercised in the adversarial suite, rather than threshold arithmetic.

## 10 Discussion

### 10.1 Limitations

The governance engine evaluated here is open source and byte-identical to the engine AEGIS runs, so its determinism, threshold-sweep, adversarial, confidence-cap, and safety-analysis results are openly reproducible by a one-command regeneration (Section 9.1); only the host-specific latency figures and the proprietary planning agent that generated the trajectory corpus are implementation-specific (Data Availability Statement).

LATTICE addresses the architectural basis for authorization but does not guarantee model correctness. Probabilistic components (the LLM-based planning agent) may produce unexpected outputs. The architecture's value lies in constraining these outputs to acceptable bounds through deterministic governance evaluation.

The assurance properties assume correct implementation. Bugs in the governance engine, execution gate, or audit system could create vulnerabilities. The empirical evaluation (Section 9) validates the reference implementation against these properties, but does not constitute a formal proof of correctness. Thorough testing, including red-team exercises against the governance boundary, remains essential for production deployments.

The confidence scoring model relies on the planning agent's ability to accurately self-assess across five component dimensions. If the planning agent systematically overestimates its confidence (a form of miscalibration), the governance system could permit more autonomous operation than warranted; recent surveys confirm that LLMs frequently exhibit systematic overconfidence across diverse tasks (Geng et al., 2024). LATTICE mitigates this through the policy-derived confidence cap and fail-closed design, with independent policy-constraint evaluation that blocks violations regardless of the reported score (Section 5.6). The confidence score should therefore be understood as a governance triage heuristic, routing actions to the appropriate oversight level, rather than a validated estimator of real-world operational risk. Future work should investigate calibration techniques for confidence component estimation, including empirical validation of the correlation between confidence scores and actual action outcomes.

The real-LLM planner-invariant safety evaluation (Section 9.11) carries validity caveats stated with the result there: its ground truth is a *specification-derived rule oracle* rather than a field-measured outcome; *autonomy is not assessed*, because the policy's required structured prerequisite token went unmet and essentially every action was escalated or blocked, so the run establishes planner-invariant safety rather than autonomous throughput; the independent LLM judges agreed only weakly with the oracle (Cohen κ from −0.05 to 0.28) and are reported only as a low reliability bound; and the policy-derived confidence cap was an *unexercised backstop* in this corpus. A human-adjudicated sample and a higher-autonomy, deployment-faithful configuration would further strengthen construct and external validity. LATTICE governs *structured* action attributes; free-text payload injection into natural-language content is out of model and is the responsibility of an upstream input-sanitization layer.

### 10.2 Deployment considerations

Practical deployment of LATTICE introduces considerations beyond the architectural specification.

#### 10.2.1 Performance overhead

The governance evaluation path adds latency to every action. The policy engine uses pure functions with no external dependencies, so evaluation latency is dominated by the number of policy rules and the complexity of confidence component computation. On the disclosed host (Section 9.8), policy evaluation completes in single-digit microseconds (p50 ≈ 6 μs), and full gated enforcement including hash verification and signed audit I/O adds approximately 0.7 ms at p50; ECDSA sign/verify add roughly 20–40 μs per audit record. These figures are host-specific. For time-critical applications, the governance evaluation can be pipelined with action preparation to hide latency.

#### 10.2.2 Policy management

Organizations deploying LATTICE must develop and maintain policy bundles that accurately encode their governance requirements. Policy authoring errors (e.g., overly permissive rules, missing constraints) can undermine the architecture's guarantees. Version-controlled policy bundles with review workflows, automated testing against known-good and known-bad action bundles, and staged deployment (shadow mode before enforcement) reduce this risk.

#### 10.2.3 Operator integration

The HITL escalation mechanism requires integration with operator workflows. Escalation fatigue (too many ESCALATE verdicts overwhelming operators) degrades the quality of human oversight Tariq et al. (2025). The converse risk is equally significant: automation complacency, where operators routinely approve escalated actions without adequate scrutiny. Parasuraman and Manzey (2010) show that complacency and automation bias arise from attentional reallocation when operators develop high trust in automated systems, leading to degraded monitoring of automation outputs, precisely the failure mode that LATTICE's HITL band is intended to prevent. Threshold tuning (Section 5.4) and escalation batching can manage operator workload, but the optimal configuration is deployment-specific and requires empirical calibration.

### 10.3 Alignment with U.S. AI governance frameworks

LATTICE is an architectural control layer rather than a policy regime, but its mechanisms map directly onto the principal U.S. federal AI governance instruments. The most durable of these is the NIST AI Risk Management Framework (Tabassi, 2023), whose four functions LATTICE operationalizes. **Govern** is realized as policy-as-code: organizational risk decisions are encoded in signed, versioned policy bundles and enforced deterministically rather than left to operator discretion. **Map** corresponds to the authorization-provenance and scope records that situate each action in its approved operational context. **Measure** is served by the confidence model, the policy-derived cap, and the auditable verdict stream, which quantify and bound action risk before execution. **Manage** is embodied in the escalation, kill-switch, and override mechanisms that allocate human oversight to residual risk. The NIST Generative AI Profile (NIST, 2024), which extends the framework to generative systems, emphasizes precisely the failure modes LATTICE targets, confabulation, unsafe autonomous action, and lack of traceability, by treating the planner as untrusted and gating its outputs.

At the federal-policy level, the current posture, Executive Order 14179 (The White House, 2025a) and the 2025 AI Action Plan (The White House, 2025b), frames governance as an enabler of accelerated, trustworthy adoption rather than a brake on it, which is precisely LATTICE's thesis that authorization is an engineering property. OMB Memorandum M-25-21 (Office of Management and Budget, 2025), which governs federal agency use of AI, requires minimum risk-management practices for “high-impact” AI: pre-deployment testing, ongoing monitoring, human oversight, and documentation. LATTICE supplies architectural substrate for each, pre-deployment property tests and the adversarial suite, the append-only audit chain for monitoring, the HITL/escalation path for oversight, and the per-claim evidence map for documentation. We do not claim that LATTICE discharges any legal obligation; rather, it provides verifiable mechanisms an organization can cite when demonstrating conformance. Because these instruments are voluntary or executive and subject to revision, the mapping is to their stable substance, testing, oversight, and traceability, not to any single directive.

### 10.4 Responsible and explainable AI

LATTICE incorporates responsible-AI (RAI) and explainable-AI (XAI) principles, but at the level of *governance decisions* rather than the planner's internal cognition, a distinction the architecture makes deliberately. On responsibility, the architecture operationalizes accountability (every verdict, escalation, and execution is cryptographically attributable to a principal through the audit chain and authorization provenance), human oversight (the confidence cap and ESCALATE path keep a human in the loop for high-consequence actions), and safety and security (fail-closed enforcement, the adversarially-tested boundary, and the kill switch). On explainability, each governance decision carries a machine-checkable rationale: the verdict, the effective confidence *c*_eff_, and an explicit set of reason codes (for example CONFIDENCE_CAP_APPLIED or POLICY_RULES_INVALID) that state *why* an action was allowed, blocked, escalated, or capped. Because policy is code and evaluation is deterministic, these explanations are faithful by construction: they are the actual decision procedure, not a post-hoc approximation of it.

What LATTICE deliberately does *not* attempt is to explain the planner's internal reasoning. The opacity of LLM cognition is the premise of the design (Section 1): rather than certifying *why* a probabilistic model proposed an action, LATTICE makes the *governance* of that action transparent and enforceable. This bounds the XAI claim honestly: LATTICE offers decision-level, not model-level, explainability, and it is exactly this separation that lets the governance layer remain auditable even when the planning model is not.

### 10.5 Reference implementation

A reference implementation (AEGIS) provides laboratory validation of LATTICE properties. AEGIS implements the Team tier (4 Grid Cells) with relaxed confidence thresholds (θ_auto_=0.85, θ_hitl_=0.65, θ_esc_=0.45) that widen the autonomous operation envelope relative to the architecture defaults (0.90/0.60), appropriate for offensive security operations, with all kill-switch channels enabled and maximum audit verbosity. The AEGIS backend integrates the three governance-stack packages, MANDATE (Calboreanu, 2026c), the LATTICE engine evaluated here, and TRACE (Calboreanu, 2026d) (Section 9.1). Together they total 996 passing tests in the proprietary monorepo's internal continuous-integration suite (an internal figure, distinct from the released LATTICE engine's 367-test public suite reported in Section 9), completing in approximately 6.5 s. The present paper concerns only the authorization layer; the runtime execution layer (Calboreanu, 2026d) and the cross-model audit method used in our evaluation (Calboreanu, 2026a) are reported in cited companion work. Section 9 presents detailed empirical results from this implementation.

**Code Availability**. The LATTICE architecture specification, policy schemas, formal property definitions, and test vectors are available at https://github.com/calboreanu/LATTICE under the Apache 2.0 license. The public repository includes schemas, sample policy bundles, test vectors, and a reference pipeline. AEGIS, the full reference implementation, is proprietary; however, the LATTICE architecture itself is fully specified and can be reimplemented using open-source components.

### 10.6 Second-domain instantiation: critical infrastructure

To test whether LATTICE governance primitives transfer beyond the security operations domain, we instantiated a complete policy bundle for a second domain (scheduled switching operations at a regional electric utility) and executed it on the public LATTICE reference pipeline.

#### 10.6.1 Domain mapping

The electric utility scenario models an autonomous agent managing scheduled breaker and switching operations at a single substation (SUB-47) during a planned maintenance window. Domain-specific adaptations required only three categories of change: (1) scope constraints were redefined from network targets to named substation equipment identifiers; (2) tool classes were remapped from offensive security operations (RECON, SCAN, EXPLOIT) to utility operations (MONITOR, SWITCH, PROTECT); and (3) confidence component semantics were reinterpreted for the new domain (e.g., “tool specificity” became “equipment-to-procedure match,” “constraint completeness” became “safety prerequisite satisfaction”). The core governance primitives (policy-as-code enforcement, deterministic verdict routing, confidence-based escalation, and cryptographic audit trails) required no modification.

#### 10.6.2 Threshold calibration

The CI domain uses conservative thresholds derived from the Solo tier (θ_high_ = 0.95, θ_low_ = 0.75; the low bound is tightened from the 0.70 Solo default in Table 6), reflecting the lower risk tolerance of electric utility operations compared to offensive security. The narrower HITL band pushes more actions to human review, consistent with the operational culture of electric utility switching, where independent verification is standard practice.

#### 10.6.3 Test cases and results

Three action bundles were constructed to exercise the full verdict space:

1. **Routine breaker opening** (all prerequisites met, composite confidence 0.95): the policy engine returned ALLOW with confidence 95.0 and the execution gate passed the action. In-scope, fully verified operations proceed autonomously when confidence meets the elevated threshold.
2. **Relay protection setting change** (incomplete contingency evidence, composite confidence 0.76, irreversibility flag set): the policy engine returned ESCALATE with reason code IRREVERSIBLE_ ACTION_ REQUIRES_ HUMAN_ REVIEW; the execution gate correctly blocked the action pending human approval. The irreversibility flag, encoded as a scope tag matched by a SCOPE_TAG_DENYLIST rule with ESCALATE effect, forces human review regardless of confidence. Domain-specific safety constraints (here, the principle that protection setting changes require operator confirmation) are enforceable through existing LATTICE rule primitives without engine modification.
3. **Out-of-scope target** (different substation SUB-52, composite confidence 0.92): the policy engine returned BLOCK with reason codes TARGET_ OUT_ OF_ SCOPE and TARGET_ NOT_ IN_ WORK_ ORDER; the execution gate blocked the action. Despite the planner's high self-reported confidence, the deterministic policy constraint evaluation overrode the confidence score, the same invariant that prevents scope violations in the security domain.

All three verdicts matched expectations. The full audit trace for Bundle 1 (the ALLOW case), including SHA-256 governed-unit hashes and hash-chained audit records, verified that the cryptographic audit chain operates identically across domains. The CI policy bundle, action bundles, and test outputs are deposited in the public repository.

#### 10.6.4 Rule-engine expressivity gaps observed during this instantiation (since closed)

Two domain-specific constraints identified in the scenario specification, maintenance time-window enforcement and prerequisite-check gating (e.g., verifying upstream isolation before downstream switching), were not expressible as native rule types in the policy-engine version used for this instantiation. At that stage, these constraints would have required either engine extension or an external pre-gate validation layer for production CI deployments. The three test cases produce correct verdicts without these rule types because the relevant safety state is encoded in the confidence components (affecting the composite score) and in scope tags (triggering the irreversibility rule); however, a production deployment should not rely on confidence-mediated enforcement for hard safety prerequisites.

#### 10.6.5 Implications

This instantiation is a *feasibility illustration* of cross-domain transfer rather than a claim of broad generalization: it shows the governance core can be reparameterized for an additional domain while revealing concrete rule-expressivity gaps. The engine evaluated in Section 9 has since closed those gaps, maintenance time-windows and prerequisite checks are now native rule types (TIME_WINDOW, PREREQUISITE), exercised by dedicated tests (Section 9.4), and the real-LLM study (Section 9.11) additionally exercises trajectories spanning multiple operational domains. LATTICE's domain-agnostic governance core (the execution gate, policy engine, confidence routing, and audit chain) transferred without code changes. Only the policy bundle contents (scope constraints, tool authorizations, threshold values) and the semantic interpretation of confidence components required domain-specific authoring. The rule engine limitations noted above identify a concrete extension point: domain-specific constraint types beyond target/tool allowlists and scope-tag matching. This separation supports the architectural design goal articulated in Section 4: governance mechanisms that are parameterized by, but not coupled to, the operational domain.

### 10.7 Future work

Three directions extend the current contribution:

#### 10.7.1 Scaled empirical validation

The current evaluation validates LATTICE properties at the unit and integration level. Large-scale validation, including red-team testing against the governance boundary with adversarial prompting, sustained operation over 100,000+ action evaluations, and multi-cell coordination stress testing, would strengthen the empirical basis. Rabanser et al. (2026) propose twelve metrics decomposing agent reliability along four dimensions, consistency, robustness, predictability, and safety, and show that capability gains do not automatically yield reliability improvements; their framework provides a principled basis for evaluating LATTICE deployments beyond task-level accuracy.

#### 10.7.2 Domain generalization

The reference implementation targets autonomous security operations. Adapting LATTICE to other high-consequence domains (medical diagnosis, financial trading, critical infrastructure control) requires domain-specific policy bundles, confidence component definitions, and threshold calibration. Recent domain-specific governance research reinforces both the need and the feasibility of such adaptation: in the military domain, Sahoo (2026) proposes the Agentic Military AI Governance Framework (AMAGF) with preventive, detective, and corrective governance pillars and a real-time Control Quality Score, architectural concerns that align closely with LATTICE's confidence-based escalation and kill-switch mechanisms. In healthcare, Alelyani (2025) validates a ten-dimension governance framework for autonomous clinical systems, identifying human–AI collaboration and regulatory compliance as critical dimensions that map directly to LATTICE's HITL escalation and compliance evidence generation. In finance, Kurshan et al. (2025) decompose oversight into four layers of regulatory blocks, from self-regulation modules embedded beside each model to systemic market-level monitoring, while Gong (2026) argues that the systemic implications of AI agents in financial markets depend less on model intelligence than on how agent architectures are distributed, coupled, and governed, proposing bounded autonomy as the near-term equilibrium. For critical infrastructure, Sharma and Pursiainen (2026) outline four oversight modes for embodied AI mapped to sectors by task complexity, risk level, and consequence severity, drawing on the EU AI Act and ISO safety standards, a governance stratification that parallels LATTICE's confidence-tier mechanism; this academic work is complemented by joint government guidance from nine agencies establishing four principles for secure AI integration in operational technology environments (Cybersecurity and Infrastructure Security Agency et al., 2025). The architecture's domain-agnostic core (the execution gate, audit chain, and verdict semantics) should transfer; the domain-specific components (policy rules, confidence factors, escalation criteria) require new development informed by these emerging domain frameworks.

#### 10.7.3 Formal verification

While the empirical evaluation demonstrates correct behavior across tested inputs, formal verification using model checking or theorem proving would provide stronger guarantees. Properties amenable to formal verification include governance completeness (every action receives a verdict), audit chain integrity (the hash chain is unforgeable given A1 and A2), and non-bypassability (no code path exists from planning to execution that does not traverse the gate).

## 11 Conclusion

LATTICE provides an architectural foundation for deploying autonomous AI agents in environments where organizational authorization requires verifiable governance. By reframing authorization from “do we trust this AI?” to “do we trust this architecture?”, LATTICE enables engineering validation of governance guarantees that hold under explicit, stated assumptions.

The 1+3 Grid Cell pattern enforces separation between planning, execution, and governance functions, removing, by construction, the conflict of interest inherent in systems where agents judge their own compliance. Policy-as-code enforcement ensures deterministic verdicts. Gated execution paths, under the trusted-infrastructure assumptions A1–A5, prevent unauthorized actions. Cryptographic audit trails preserve complete decision provenance. Confidence-based escalation routes human oversight according to a policy-derived risk signal. Hard safety prerequisites are enforced as deterministic rules (for example, maintenance-window and prerequisite checks) rather than resting on confidence components.

Empirical evaluation against the AEGIS reference implementation provides evidence for these properties within the tested configuration space: correct verdicts across all 43 determinism test cases, no unauthorized executions observed across 22 non-bypassability tests, tamper-evident audit chains with cryptographic verification, and successful end-to-end governance chain validation across a 36-step integration scenario spanning specification, authorization, and execution control. These results bound the evaluated attack surface and configuration space; they do not constitute proof of universal correctness. A pre-specified study across four frontier planner families further showed planner-invariant safety: no oracle-flagged unsafe action was admitted (false-allow 0.0, recall 1.0), achieved at a conservative operating point at which no action was auto-allowed. A live closed-loop run separately authorized and executed real operating-system actions with zero unsafe executions over a 10,000-action burn-in (Section 9.12), demonstrating the authorize-and-execute path; autonomous throughput on real-agent workloads at scale remains future work.

These mechanisms transform autonomous AI deployment from an act of faith into an engineering discipline. LATTICE does not solve AI alignment or guarantee model behavior. It provides the architectural substrate that makes authorization decisions tractable: organizations can verify scope enforcement, audit policy compliance, test escalation thresholds, and demonstrate halt mechanisms. For defense organizations, critical infrastructure operators, and regulated industries seeking to adopt autonomous AI capabilities, this engineering basis for authorization represents a necessary step toward responsible deployment.

## Data Availability

The LATTICE governance engine, architecture specification, policy schemas, sample policy bundles, test vectors, and the reproducibility drivers are publicly available at https://github.com/calboreanu/LATTICE under the Apache 2.0 license; the engine evaluated here is byte-identical to the engine used by the AEGIS reference implementation. A complete evaluation package, harnesses, raw results, the pre-specified safety-evaluation protocol and corpus, the latency measurements, the integrity manifest, and a separate replication pipeline, distinct from the harness that generated the results, that recomputed the headline statistics from the raw artifacts under the author's direction, is provided as an archived artifact, and a single documented command regenerates every open-tier result, and each reported number is mapped to its producing artifact through a claims-to-evidence index included in the package. The proprietary AEGIS planning agent and the latency measurement host are implementation-specific: latency figures are reported at the host disclosed in Section 9 and are not community-reproducible, whereas the determinism, threshold-sweep, adversarial, confidence-cap, and safety-analysis results reproduce from the public artifact. No benchmark artifacts are withheld pending acceptance. The Supplementary material accompanying this article provides the reference and data tables (Tables S1–S34), including the per-planner safety ladder, the adversarial and pre-specified hypothesis tests, the judge-reliability and determinism data, the source-status classification of comparison systems, and the live-execution and soak results.
