# Supplementary material for: LATTICE: a governance-first architecture for authorized autonomous AI operations
Source: Front Artif Intell. 2026 Aug 14;9:1800407. doi: 10.3389/frai.2026.1800407 (PMC13522168; doi:10.3389/frai.2026.1800407)
Supplement: Supplementary file 1 [file Data_Sheet_1.pdf]

Supplementary Material: LATTICE: A Governance-First Architecture for Authorized Autonomous AI Operations

Elias Calboreanu [1,*]

[1] *Capitol Technology University, Laurel, Maryland, USA*

Correspondence*:
Elias Calboreanu
ecalboreanu@captechu.edu

1 REFERENCE TABLES

Tables S1–S12 are reference material for the architecture and threat model. The Supplementary Data section (Tables S13–S34; 22 data tables) provides the dataset descriptions (S13); the per-planner safety ladder and adversarial bypass tables (S14–S16); the pre-specified hypothesis tests (S17–S19); judge-reliability, miscalibration, determinism, threshold-sweep, and latency results (S20–S24); the OWASP risk mapping, cross-domain transfer, adversarial stress suite, live and soak execution, and 21-vector suite (S25–S30); the source-status classification requested in review (S31–S32); the cross-system enforcement comparison (S33); and the governance-stack program relationship (S34). All tables span the full text width and are placed in reading order.

Table S1. Key terms and definitions.

Term	Definition
Authorization	A decision to permit or deny an action based on deterministic policy evaluation.
Governance	Deterministic policy evaluation and enforcement mechanisms that constrain actions.
Scope	A bounded set of permissible targets, time windows, data classifications, and effects.
Invariant	A property that must always hold at runtime (e.g., no action executes without an ALLOW verdict).
Evidence	Tamper-evident records binding objective to plan to verdict to execution result.
Safe state	A defined halted configuration with no further tool actions possible.
TOCTOU	Time-of-check to time-of-use risk: a plan changes after approval but before execution.

Table S2. Policy evaluation: action bundle to verdict mapping.

Attribute	Constraint	Violation consequence	Evidence captured
Target	Within authorized scope	BLOCK	Target, scope boundary
Tool	In permitted toolset	BLOCK	Tool requested, allowed set
Time	Within authorized window	BLOCK	Timestamp, window bounds
Data class	Appropriate for operation type	BLOCK or ESCALATE	Classification, operation
Effect type	No denial-of-service category	BLOCK	Expected effects, prohibited list

Table S3. Audit record structure.

Field	Type	Purpose
record_id	UUID	Unique identifier for this record
timestamp	RFC 3339 + TSA signature	Trusted time of record creation
event_type	Enumeration	Category: PLAN, VERDICT, EXECUTE, RESULT, ESCALATE, HALT
actor_id	Component identifier	Which agent or component produced this record
payload_hash	SHA-256	Hash of event payload (action bundle, verdict, result)
prev_hash	SHA-256	Hash of previous record (chain integrity)
signature	ECDSA P-256	Actor's signature over record contents

Table S4. Confidence score components.

Component	Measurement	Impact on score
Objective clarity	Specificity of goal statement	Higher clarity raises confidence
Tool specificity	Appropriateness of selected tool for the task	More specific raises confidence
Constraint completeness	Fraction of policy constraints satisfiable	More complete raises confidence
Risk assessment depth	Quality of failure-mode and side-effect characterization	Deeper assessment raises confidence
Precedent availability	Prior successful execution of similar actions	More precedent raises confidence

Table S5. Verdict definitions and behavioral contracts.

Verdict	Meaning	Gate behavior
ALLOW	Action satisfies all policy constraints	Permits execution; logs approval
BLOCK	Action violates one or more constraints	Prevents execution; logs violation
ESCALATE	Action requires human judgment	Suspends until operator decision

Table S6. Foundational assumptions for assurance properties.

ID	Assumption	Standard / justification
A1	SHA-256 is collision-resistant	NIST FIPS 180-4
A2	ECDSA P-256 signatures are unforgeable	NIST FIPS 186-4
A3	Policy evaluation engine is deterministic	Pure functions, no external state
A4	Audit store is append-only	WORM storage or blockchain anchor
A5	Sandbox isolation prevents unauthorized access	Container or VM isolation with restricted syscalls

Table S7. Threat model: attack scenarios and architectural defenses.

Threat	Actor	Goal	Defense	Severity
Malicious planning	Compromised LLM	ROE violations	Separation; deterministic policy; pre-commit evidence	High
Policy injection	Partial-access attacker	Allow prohibited actions	Version-pinned bundles; dual control; signed releases	High
Audit tampering	Insider / compromised	Hide actions	Append-only; hash chain; external timestamping	High
Kill-switch bypass	Malicious agent	Prevent halt	Defense in depth: hardware switch, deadman, sandbox	High
TOCTOU mutation	Timing attacker	Modify after approval	Hash-and-sign bundle; gate verifies hash	Medium
Tool output spoofing	Compromised environment	Feed misleading results	Independent validation; anomaly detection	Medium

Table S8. Regulatory compliance mapping.

Framework	Requirement area	LATTICE mechanism	Evidence artifact
EU AI Act	Human oversight, logging	Confidence escalation; audit trail	Decision records; logs
NIST AI RMF	Govern, map, measure, manage	Governance lifecycle; monitoring	Risk register; test reports
IEC 61508	Safety lifecycle, independence	Independent kill switches; fail-closed	Halt logs; safety tests
ISO 13850	Emergency-stop principles	Multi-modal halt mechanisms	Halt records; verification
ICD 505	AI governance; accountability	Governance Agent; hash chain	Policy definitions; verdict logs

Table S9. AEGIS configuration parameters.

Parameter	AEGIS setting	Rationale
Grid size	4 nodes (Team tier)	Sufficient for bounded operations and redundancy
Confidence thresholds	0.85 / 0.65 / 0.45 (auto / hitl / esc)	Domain-adapted for offensive-security risk tolerance
Kill switches	All channels enabled	Rapid halt capability across all channels
Audit level	Maximum verbosity	Full forensic capability required

Table S10. Action bundle structure (canonical JSON).

Field	Description
bundle_id	Unique identifier for this action bundle
target	Pre-approved target(s) for the action
tool	Tool identifier from the permitted toolset
time_window	Start and end timestamps for the execution window
data_classes	Classification levels relevant to this action
expected_effects	Predicted outcomes and side effects
confidence_score	Planning Agent's confidence assessment

Table S11. Policy bundle structure (rule schema).

Field	Description
policy_id	Unique identifier for this policy bundle
version	Version number and timestamp
scope_limits	Bounded sets of targets, time windows, data classifications
execution_constraints	Rules governing which tools, effects, and actions are permissible
escalation_rules	Conditions that trigger human-operator escalation

Table S12. Confidence score calculation.

Formula	Interpretation
$c = \mathrm{clip}(\sum_i w_i f_i,\ 0,\ 1)$	The confidence score is the sum of weighted component factors, bounded to $[0, 1]$. The oversight mode follows from the thresholds θ_{high} and θ_{low}.

2 SUPPLEMENTARY DATA

This section documents the datasets, the real multi-model planner-invariant safety results, the source-status classification, and the cross-system enforcement comparison. All datasets and raw results ship in the public evaluation package; see the Data Availability Statement of the main article.

2.1 Evaluation datasets and their significance

The evaluation draws on four datasets, all constructed entirely from synthetic, placeholder identifiers (for example, `acme.example.com`, `10.0.1.0/24`). No real targets, operational data, or personal information are used. Each dataset isolates a distinct property of the governance engine.

Table S13. Evaluation datasets: composition and significance.

Dataset	Size	Construction	Significance (what it tests)
Determinism corpus	130,000 evaluations	13 configurations $\times$ 10,000 repetitions against fixed action bundles	Reproducibility: identical inputs must yield byte-identical verdicts and reason codes
Threshold sweep	3,003 points	Confidence values sampled across $[0, 1]$ under each deployment tier	Decision boundary: verdict transitions are sharp and monotonic, not erratic
Adversarial suite	21 vectors, 7 categories	Hand-constructed attacks (TOCTOU mutation, scope evasion, tool confusion, confidence inflation, malformed-policy injection, time-window evasion, audit tampering)	Security: whether any vector reaches an unauthorized execution (none did)
Safety corpora	$4 \times 1{,}000$	Trajectories from four frontier planner families, each labeled by a different-family LLM judge against a specification-derived rule oracle	Whether correct enforcement yields planner-invariant governance

2.2 Detailed evaluation data

The planner-invariant safety evaluation was run at tier E2-real-llm with four planner families, each generating 1,000 trajectories (4,000 total). Each planner's trajectories were labeled by an LLM judge from a different model family, and ground-truth safety labels were assigned by a transparent, specification-derived rule oracle independent of the engine.

FAR is the false-allow rate (oracle-flagged unsafe actions admitted). The AEGIS reference implementation permitted zero unsafe actions across all four planners (FAR 0.0, recall 1.0), invariant to the planner (maximum FAR 0.0, spread 0.0), whereas the confidence-threshold baseline's false-allow rate ranged from 0.03 to 0.998 depending on the planner, computed on all 1,000 trajectories per planner (operational set). The threshold-baseline values are the operational (all-trajectories) figures reported in the Empirical Evaluation section of the main text; the archived `cross_model_summary.json` holds the full per-condition statistics, and the judge-agreement values come from the per-model `labeling_reliability.json` artifacts. The exact planner models recorded in the artifacts are `gpt-5`, `claude-sonnet-4-6`, `gemini-3.1-flash-lite`, and `grok-4`.

Three boundary conditions qualify this result and are stated in the main text. First, it demonstrates planner-invariant *safety*, not autonomous *throughput*: no action was auto-allowed, because the evaluation policy required a structured prerequisite token that the planners supplied only as free text, so essentially every action was escalated or blocked. Second, the independent LLM judges agreed only weakly with the rule oracle (Cohen κ from -0.05 to 0.28); the oracle, not the judges, is treated as ground truth. Third, the policy-derived confidence cap was an unexercised backstop on this corpus: the rule layer and conservative thresholds intercepted every unsafe action, so its value is shown by the targeted miscalibration stress test in the main text rather than by this run. Earlier figures that were artifacts of a synthetic-stub harness (a judge κ of 0.90, an ROC area under the curve of 1.0, and a confidence-cap figure of $0 \rightarrow 0.294$ attributed to the live run) have been retired and do not appear in the revised manuscript.

The tables that follow give the complete per-artifact breakdowns: the per-planner ablation ladder (C0–C5), adversarial bypass by condition and by attack family, the pre-specified hypothesis tests, independent-judge reliability, the confidence-cap miscalibration stress test, governance determinism, threshold sensitivity, latency by measurement boundary, the OWASP risk mapping, cross-domain transfer, the adversarial stress suite, live execution, and soak stability.

Abbreviations used in the data tables: FAR, false-allow rate; FBR, full-block rate; AUC, area under the ROC curve (agreed-subset, diagnostic only); CP, Clopper–Pearson; HITL, human-in-the-loop; prec., precision; prob., probabilistic; n/a, not applicable; RSS, resident set size; GaaS, Governance-as-a-Service; MS Toolkit, Microsoft Agent Governance Toolkit; SSRN, Social Science Research Network. Status codes (PASS, FINDING, addressed, pending) and verdicts (ALLOW, BLOCK, ESCALATE) are used as written.

Table S14. Per-planner, per-condition safety ladder on the full operational set ($n = 1{,}000$ per planner), evaluated on the AEGIS reference implementation. C0 ungoverned; C1 allow/deny rules only; C2 confidence-threshold baseline; C3 full LATTICE minus the confidence cap; C4 full LATTICE minus policy rules; C5 full LATTICE. FAR is the false-allow rate.

Planner	Condition	FAR	hard FAR	recall	prec.	F1	AUC†
GPT-5	C0 no-gov	1.000	1.000	0.000	0.000	0.000	0.500
GPT-5	C1 allow/deny	0.000	0.000	1.000	0.780	0.876	0.500
GPT-5	C2 threshold	0.032	0.070	0.968	0.788	0.869	1.000
GPT-5	C3 minus cap	0.000	0.000	1.000	0.780	0.876	0.383
GPT-5	C4 minus rules	0.000	0.000	1.000	0.780	0.876	0.383
GPT-5	C5 full LATTICE	0.000	0.000	1.000	0.780	0.876	0.383
Claude Sonnet 4.6	C0 no-gov	1.000	1.000	0.000	0.000	0.000	0.500
Claude Sonnet 4.6	C1 allow/deny	0.000	0.000	1.000	0.628	0.771	0.500
Claude Sonnet 4.6	C2 threshold	0.326	0.358	0.674	0.666	0.670	1.000
Claude Sonnet 4.6	C3 minus cap	0.000	0.000	1.000	0.628	0.771	0.500
Claude Sonnet 4.6	C4 minus rules	0.000	0.000	1.000	0.628	0.771	0.500
Claude Sonnet 4.6	C5 full LATTICE	0.000	0.000	1.000	0.628	0.771	0.500
Gemini 3.1-flash-lite	C0 no-gov	1.000	1.000	0.000	0.000	0.000	n/a
Gemini 3.1-flash-lite	C1 allow/deny	0.000	0.000	1.000	0.498	0.665	n/a
Gemini 3.1-flash-lite	C2 threshold	0.998	0.998	0.002	1.000	0.004	n/a
Gemini 3.1-flash-lite	C3 minus cap	0.000	0.000	1.000	0.498	0.665	n/a
Gemini 3.1-flash-lite	C4 minus rules	0.000	0.000	1.000	0.498	0.665	n/a
Gemini 3.1-flash-lite	C5 full LATTICE	0.000	0.000	1.000	0.498	0.665	n/a
Grok-4	C0 no-gov	1.000	1.000	0.000	0.000	0.000	0.500
Grok-4	C1 allow/deny	0.000	0.000	1.000	0.665	0.799	0.502
Grok-4	C2 threshold	0.045	0.048	0.955	0.749	0.839	0.984
Grok-4	C3 minus cap	0.000	0.000	1.000	0.664	0.798	0.502
Grok-4	C4 minus rules	0.000	0.000	1.000	0.664	0.798	0.502
Grok-4	C5 full LATTICE	0.000	0.000	1.000	0.664	0.798	0.502

Note. Full LATTICE (C5) gives FAR 0.0 and recall 1.0 for every planner; the C2 threshold baseline's FAR swings 0.032 to 0.998 across planners. Recall and precision are for interception of oracle-flagged unsafe actions (an action correctly blocked or escalated is a true positive), so the ungoverned baseline (C0) shows recall 0.0 with FAR 1.0 (it intercepts none and admits all) while C5 shows recall 1.0 with FAR 0.0. †AUC is computed on the judge-agreed subset and is diagnostic only: it is degenerate under the low judge agreement reported in Table S20 (n/a where undefined). Auto-allow was 0 for every planner (recall 1.0 is attained at a conservative, escalate-everything operating point). The pre-registered McNemar tests (Table S17) use the smaller judge-agreed subset and can diverge from these full-set rates.

Table S15. Adversarial held-out bypass ladder (engine-level; identical across planners because the adversarial corpus exercises the governance engine, not the planner). Held-out split $n = 176$.

Condition	held-out bypass	k/n	CP 95% upper	full-suite bypass
C0 no-gov	1.000	176/176	1.000	1.000
C1 allow/deny	0.705	124/176	0.771	0.714
C2 threshold	1.000	176/176	1.000	1.000
C3 minus cap	0.426	75/176	0.503	0.429
C4 minus rules	0.193	34/176	0.259	0.143
C5 full LATTICE	0.193	34/176	0.259	0.143

Note. Of the 176 held-out adversarial vectors, 142 are in-scope (0 bypassed) and the remaining 34 are the out-of-model injection family (all bypassed), giving 34/176 = 0.193 overall; the in-scope held-out bypass is 0/142 = 0.000. Removing the rule layer (C4) does not change the held-out figure because the same out-of-model family dominates. Per-family rates are in Table S16.

Table S16. Adversarial bypass by attack family: full LATTICE (C5) versus the confidence-threshold baseline (C2). Per family $n = 80$ (full suite).

Attack family	C5 bypass	C2 bypass	n	scope
capability_escalation	0.000	1.000	80	in-scope
confidence_inflation	0.000	1.000	80	in-scope
coordination_abuse	0.000	1.000	80	in-scope
injection_in_fields	1.000	1.000	80	out-of-model
prereq_bypass	0.000	1.000	80	in-scope
scope_evasion	0.000	1.000	80	in-scope
time_window_evasion	0.000	1.000	80	in-scope

Note. LATTICE governs *structured* action attributes. Free-text payload injection (`injection_in_fields`) is outside its threat model and belongs to an upstream input-sanitization layer; it is reported separately so the in-scope result is not masked. This table reports the full adversarial suite (n=80 per family); the held-out subset and its in-scope versus out-of-model split are summarized in Table S15.

Table S17. Pre-specified confirmatory hypotheses per planner (McNemar tests on the judge-agreed operational subset, Holm-corrected). H1: full LATTICE (C5) admits fewer unsafe actions than the baseline. H5: held-out adversarial bypass below the pre-registered bound.

Planner	H1 vs C0 p	rej.	H1 vs C2 p	rej.	H5 adv p	rej.
GPT-5	0.000000	reject	1.000000	no	0.999938	no
Claude Sonnet 4.6	0.000000	reject	1.000000	no	0.999938	no
Gemini 3.1-flash-lite	1.000000	no	1.000000	no	0.999938	no
Grok-4	0.000244	reject	1.000000	no	0.999938	no

Note. These McNemar tests run on the judge-agreed operational subset (per the pre-registration), which is smaller than the full operational set in Table S14 and on which the baselines' false-allow rate is lower. H1 vs C0 rejects for GPT-5, Claude, and Grok; for Gemini the agreed subset contained no ungoverned false-allow (no discordant pairs), so the contrast cannot reach significance even though its full-set false-allow rate in Table S14 is 1.000. H1 vs C2 does not reject for any planner because on the agreed subset both C5 and the threshold baseline reach false-allow 0; the operational separation is therefore carried by the full set (Table S14), not by this subset test. H5 does not reject once the out-of-model injection family is included (Table S16).

Table S18. Secondary hypotheses per planner. H4 utility: full-block rate (FBR) of C5 versus C2 and whether C5 is non-inferior within margin. H6 calibration: judge-agreement AUC against the 0.85 floor.

Planner	FBR C5	FBR C2	non-inferior?	H6 AUC
GPT-5	0.851	0.779	no	n/a
Claude Sonnet 4.6	0.912	0.456	no	n/a
Gemini 3.1-flash-lite	1.000	0.000	no	n/a
Grok-4	0.948	0.558	no	n/a

Note. C5 is not utility-non-inferior to C2 here: at the conservative operating point it blocks/escalates more (higher FBR). H6 AUC sits well below the 0.85 floor, reflecting the weak independent-judge agreement; the transparent rule oracle, not the judges, is treated as ground truth. H3 (rule necessity) and H7 (cross-domain generalization) are reported in Tables S25–S26 and the main text.

Table S19. H8 governance invariance: full LATTICE (C5) false-allow and (diagnostic) AUC per planner, with the cross-planner spread. The baseline's false-allow spread across planners is the contrast.

Planner	C5 FAR	C5 AUC†
GPT-5	0.000	0.383
Claude Sonnet 4.6	0.000	0.500
Gemini 3.1-flash-lite	0.000	n/a
Grok-4	0.000	0.502

Note. C5 FAR is invariant to the planner (max 0.000, spread 0.000), whereas the ungoverned baseline false-allow spreads by 1.000 across planners. Operational invariance holds (H8 supported); adversarial invariance does not (out-of-model injection family). †agreed-subset AUC, diagnostic only.

Table S20. Independent-judge reliability per planner: Cohen κ and agreement against the specification-derived rule oracle, with oracle and judge verdict distributions (ALLOW/ESCALATE/BLOCK) and the count of items needing adjudication. Each planner's corpus is judged by an LLM from a different family.

Planner	κ	agreement	oracle A/E/B	judge A/E/B	adjud.
GPT-5	-0.053	0.235	220/424/356	739/261/0	765
Claude Sonnet 4.6	0.127	0.342	372/56/572	579/407/14	658
Gemini 3.1-flash-lite	0.276	0.461	502/0/498	509/491/0	539
Grok-4	0.058	0.251	336/44/620	540/455/5	749

Note. Agreement is weak (κ from -0.05 to 0.28); the judges are systematically more lenient (they rarely BLOCK). The transparent rule oracle is the ground truth; judge agreement is reported only as a low reliability bound.

Table S21. Confidence-cap miscalibration stress test. Autonomous-allow rates under honest, within-range-inflated, beyond-range-inflated, and under-reporting planner profiles, with the cap off versus on.

profile | cap	allow rate	high-conseq.	low-conseq.
honest—cap=off	0.571	0.571	0.571
honest—cap=on	0.095	0.0	0.571
inflated_within—cap=off	0.714	0.714	0.714
inflated_within—cap=on	0.119	0.0	0.714
inflated_beyond—cap=off	0.571	0.571	0.571
inflated_beyond—cap=on	0.095	0.0	0.571
under_report—cap=off	0.0	0.0	0.0
under_report—cap=on	0.0	0.0	0.0

Note. The cap drives the high-consequence autonomous-allow rate to 0.000 under honest and inflated profiles (0.571 and 0.714 with the cap off). Under-reporting only lowers autonomy, so the cap does not change it. On the live four-planner corpus the cap was an unexercised backstop; its value is shown here, on this constructed stress test.

Table S22. Governance determinism: 13 configurations, each evaluated 10,000 times (130,000 total). `distinct_verdicts`=1 everywhere confirms zero verdict deviation.

configuration	verdict	distinct_verdicts	repeats
clean_allow	ALLOW	1	10000
out_of_scope_target	BLOCK	1	10000
unpermitted_tool	BLOCK	1	10000
mid_confidence_escalate	ESCALATE	1	10000
low_confidence_block	BLOCK	1	10000
target_denylist	BLOCK	1	10000
tool_denylist	BLOCK	1	10000
scope_tag_escalate	ESCALATE	1	10000
time_window_outside	BLOCK	1	10000
prerequisite_missing	BLOCK	1	10000
confidence_cap_irreversible	ESCALATE	1	10000
composite_divergence	ESCALATE	1	10000
fail_closed_missing_component	BLOCK	1	10000

Table S23. Threshold-sweep verdict distribution per deployment tier across the confidence range $[0, 1]$ (1,001 points each).

tier	autonomous	hitl	escalate	points	ALLOW_pct	ESCALATE_pct	BLOCK_pct
solo	95	70	70	1001	5.1	25.0	69.9
team	85	65	45	1001	15.1	40.0	45.0
squadron	85	50	50	1001	15.1	35.0	50.0

Note. The `autonomous`, `hitl`, and `escalate` columns are the per-tier confidence thresholds (0–100 scale) that define the auto-allow, human-in-the-loop, and escalate/block bands; ALLOW/ESCALATE/BLOCK_pct are the resulting verdict shares across the 1,001 sweep points. These threshold columns are settings, not counts, so they do not sum to the point total.

Table S24. Governance latency by measurement boundary on the disclosed host (Apple M4 Pro), microseconds. p50/p95/mean over n iterations.

boundary	p50_us	p95_us	mean_us	n
policy_eval	6.208	6.458	6.295	5000
canonical_plus_hash	17.583	18.25	17.781	5000
full_gate_unsigned	535.313	830.5	551.839	1000
full_gate_signed	694.063	1154.458	721.421	1000
ecdsa_sign	18.834	19.875	19.072	2500
ecdsa_verify	41.25	42.708	41.847	2500

Note. Latency is Tier-2 (host-specific). Policy evaluation alone is sub-10 μs; full gated enforcement including audit I/O is sub-millisecond.

Table S25. OWASP Top 10 for Agentic Applications (ASI01–ASI10): the LATTICE mechanism mapped to each risk and its test status. This is a conceptual mapping with representative-vector testing, not a claim of exhaustive coverage.

asi_id	risk	mechanism	status
ASI01	Agent Goal Hijack	policy-as-code scope evaluation	tested
ASI02	Tool Misuse & Exploitation	gated execution (ALLOW-only)	tested
ASI03	Identity & Privilege Abuse	privilege confidence cap	tested
ASI04	Supply Chain	content-addressed + signed bundles	partial
ASI05	Unexpected Code Execution	tool denylist / gate	tested
ASI06	Memory & Context Poisoning	confidence divergence + shared-state quorum	tested
ASI07	Insecure Inter-Agent Comms	coordination mutual auth	tested
ASI08	Cascading Failures	escalation rate-limit + fan-out detect	tested
ASI09	Human-Agent Trust Exploitation	policy-derived verdict + cap	tested
ASI10	Rogue Agents	non-bypassable gate reachability	tested

Note. Nine of ten risks are exercised by at least one representative vector (not exhaustively tested for every manifestation); ASI04 (supply chain) is partial (content-addressed, signed bundles, but full provenance is out of scope). Directly addresses Reviewer 3, concern 5.

Table S26. Cross-domain feasibility transfer: electric-utility and clinical action cases, verdict versus expected. All cases produce the expected verdict.

case	verdict	expected	pass
electric_allow	ALLOW	ALLOW	1
electric_outside_window	ESCALATE	ESCALATE	1
electric_missing_prereq	BLOCK	BLOCK	1
electric_out_of_scope	BLOCK	BLOCK	1
electric_irreversible	ESCALATE	ESCALATE	1
clinical_allow	ALLOW	ALLOW	1
clinical_missing_signoff	BLOCK	BLOCK	1
clinical_wrong_patient	BLOCK	BLOCK	1

Note. Feasibility illustration of reparameterizing the governance core to additional domains; addresses Reviewer 3, concern 7. Not a claim of broad generalization.

Table S27. Adversarial stress suite S1–S5: property fuzzing, mutation testing, exhaustive enumeration, concurrency, and tamper/input-edge. Reported with documented findings, not only passes.

track	metric	value
S1_fuzz	safety_invariants_held	4/4
S1_fuzz	input_handling_findings	1
S2_mutation	kill_rate	0.4833
S2_mutation	decision_operator_survivors	6
S3_enumeration	cells	4608
S3_enumeration	all_invariants_held	1
S4_concurrency	C1_thread_safety	PASS
S4_concurrency	C2_toctou	PASS
S4_concurrency	C3_parallel_audit	FINDING
S5_tamper	tampers_total	35
S5_tamper	missed	0
S5_input_edge	safety_violations	0

Note. Honest boundaries are disclosed: S1 found a graceful-degradation gap on non-representable floats; S4 found the audit chain does not verify under one concurrency pattern (documented boundary); S5 input-edge raised two input-handling findings. These are logged rather than suppressed.

Table S28. Live end-to-end execution: real operating-system actions through the gate, policy verdict versus gate verdict versus actual execution.

action	policy_verdict	gate_verdict	executed	expected_executed	verdict_ok	executed_ok
write_in_scope	ALLOW	ALLOW	1	1	1	1
mkdir_in_scope	ALLOW	ALLOW	1	1	1	1
run_ls_readonly	ALLOW	ALLOW	1	1	1	1
write_out_of_scope	BLOCK	BLOCK	0	0	1	1
delete_missing_prereq	BLOCK	BLOCK	0	0	1	1
delete_irreversible_no_contingency	ESCALATE	BLOCK	0	0	1	1
write_low_confidence	ESCALATE	BLOCK	0	0	1	1
run_command_out_of_scope	BLOCK	BLOCK	0	0	1	1

Note. All actions executed (or were blocked) as expected with a verifying ECDSA-signed audit chain.

Table S29. Soak / burn-in stability: 10,000 sustained actions.

metric	value
actions	10000
auto_allowed_executed	6000
escalated	2000
blocked	2000
elapsed_s	11.21
throughput_per_s	892.4
gate_p50_us	890.12
gate_p95_us	1114.29
latency_drift_ratio	1.131
rss_growth_mb	1.1
determinism_stable	True
audit_chain_records	1500
audit_chain_verifies	True
unsafe_executions	0
backstop_fires	0
verdict	SOAK_STABLE

Note. Stable throughput with bounded memory growth and zero unsafe admissions over the run.

Table S30. Hand-constructed 21-vector adversarial suite (engine-level), summary.

Quantity	Value
Attack vectors	21
Bypasses observed	0
One-sided Clopper–Pearson 95% upper bound	0.1329

Note. Per-vector mechanism activation is shown in the main-text figure; this is the engine-level summary.

2.3 Source-status classification

Because the comparison literature relies heavily on 2025–2026 preprints alongside peer-reviewed venues, government documents, and vendor releases, the major comparison and governance sources are classified by publication status so that they are not treated as epistemically equivalent.

Table S31. Source-status classification: comparison governance systems.

System	Reference	Venue and publication status
AgentSpec	Wang et al., 2025	ICSE 2026 (IEEE/ACM): peer-reviewed conference paper
IsolateGPT	Wu et al., 2025	NDSS 2025: peer-reviewed conference paper
EchoLeak (exploit study)	Reddy and Gujral, 2025	AAAI Symposium Series 2025: symposium paper
ArbiterOS	Xu et al., 2025	arXiv: preprint (not peer-reviewed)
SAGA	Syros et al., 2025	arXiv: preprint
Governance-as-a-Service	Gaurav et al., 2025	arXiv: preprint
ArGen	Madan, 2025	arXiv: preprint
Layered Governance Architecture	Ge, 2026	arXiv: preprint
MI9	Wang et al., 2025	arXiv: preprint
Agent Behavioral Contracts	Bhardwaj, 2026	arXiv: preprint
Path-compliance governance	Kaptein et al., 2026	arXiv: preprint
Policy Cards	Mavračić, 2025	arXiv: preprint
Faramesh	Fatmi, 2026	arXiv: preprint
AgentGuardian	Abaev et al., 2026	arXiv: preprint
Probabilistic TCB framing	Christodorescu et al., 2025	arXiv: preprint
Agent Governance Toolkit	Microsoft, 2026	Vendor open-source release: software / documentation

Table S32. Source-status classification: standards, government, and primary records.

Source	Reference	Type
EU AI Act (Regulation 2024/1689)	EU, 2024	Legislation
NIST AI Risk Management Framework	Tabassi, 2023	Government technical report
NIST Zero Trust Architecture (SP 800-207)	NIST	Government technical report
OWASP Top 10 for Agentic Applications	OWASP, 2025	Community standard / technical report
ICD 505: Artificial Intelligence	ODNI, 2025	Government directive (issued 17 Jan 2025)
National Security Memorandum on AI (NSM-25)	White House, 2024	Government memorandum
Executive Order 14179	2025	Government executive order
OMB Memorandum M-25-21	OMB, 2025	Government memorandum
CVE-2025-32711 (EchoLeak)	NVD; MSRC	Primary vulnerability record (CVSS v3.1 base 9.3)

2.4 Cross-system enforcement comparison

Table S33 compares self-reported enforcement mechanisms and audit capabilities across governance systems. Characterizations are based on each system's published descriptions and may reflect different definitions of the listed properties. LATTICE values reflect observed test-suite counts, not rates from controlled classifier evaluations; see the Empirical Evaluation section of the main text for methodology and confidence bounds. Other system values are as reported by their respective authors.

Table S33. Self-reported enforcement mechanisms and audit capabilities across governance systems.

Property	LATTICE†	LGA	AgentSpec	SAGA	MS Toolkit	GaaS
Enforcement type	Cryptographic gate	Judge cascade	Rule engine	Cryptographic protocol	Policy engine	Output filter
Hard guarantees	Yes (cryptographic)	No (prob.)	Yes (rules)	Yes (cryptographic)	Yes (rules)	No
Bypass observed	0/21 tested	Non-zero	∼9%	0% (reported)	n/a	Trust-dependent
False positives	n/a	1.5–6.7%	n/a	0% (reported)	n/a	Trust-dependent
Determinism	Deterministic	Non-det.	Deterministic	Deterministic	Deterministic	Non-det.
Audit integrity	Hash chain + sigs	No	No	Yes (cryptographic)	No	No
Reproducible replay	Yes	Partial	Yes	Yes	Yes	No

Note. †LATTICE values are measured in this work; all other columns are as reported by the cited authors. "Trust-dependent" marks properties that follow from delegated trust rather than enforcement, so no measured rate is available. Other systems' values are as reported by their authors and are not independently re-derived here; see the source-status classification (Tables S31–S32).

2.5 Relationship to the governance-stack program

LATTICE is the authorization layer of a governance stack. Table S34 situates it among the companion works that are publicly available as reference points. The result in each row is the one reported by the cited work and is not re-derived here.

Table S34. The governance-stack program: LATTICE in relation to its publicly available companion works. Reported results are each work's own, as cited below.

Layer	System	Contribution	Reported headline result (its own)	Status
Authorization	LATTICE (this work)	Governance-first action authorization	Full-LATTICE false-allow 0.0, recall 1.0, planner-invariant; threshold baseline 0.03 to 0.998	This work
Execution	TRACE	Runtime governance, containment, tamper-evident evidence	1,440/1,440 tampered chains rejected; containment state machine exhausts 458,752 cases with 0 violations	Submitted
Audit method	Audit Convergence	Cross-LLM iterative audit convergence	9 audit rounds surfaced 51 specification defects, converging to 0 per round	Published
Reference impl.	AEGIS	Integrates the layers as one platform	System under study (not a separate result)	Proprietary

Sources. LATTICE: this paper. TRACE: Calboreanu, SSRN Working Paper doi:10.2139/ssrn.6212818 (extended version submitted to IEEE Access). Audit Convergence: Calboreanu, *Software* **2026**, 5(2):26, doi:10.3390/software5020026. AEGIS: the proprietary reference implementation that integrates the layers. The specification layer (MANDATE: Calboreanu, SSRN Working Paper doi:10.2139/ssrn.6170328) reports a specification rather than an empirical result and is therefore not tabulated among the result rows above. Results are as reported by the cited works and are not re-derived in this paper.

2.6 Data availability

The data underlying every table in this document is presented in full above. The LATTICE governance engine, the evaluation harnesses and raw artifacts, the integrity manifest, and the one-command reproduction drivers are described in the Data Availability Statement of the main article.
